# Quantifying carbon stock and tree community composition in tropical forests through combining satellite and UAV analyses

**DOI:** 10.1038/s41598-025-34938-9

**Published:** 2026-01-23

**Authors:** Kotaro Komatsu, Ryuichi Takeshige, Masanori Onishi, Shogoro Fujiki, Nobuo Imai, Kazuki Miyamoto, Shin-ichiro Aiba, Kanehiro Kitayama, Sandy Tze Lui Tsen, Reuben Nilus, Joel Dawat, Joan Pereira, Yusuke Onoda, Ryota Aoyagi

**Affiliations:** 1 Graduate School of Agriculture, Kyoto University, Oiwake-cho, Kitashirakawa, Sakyo- ku, Kyoto, 606-8502 Japan; 2 Osaka Metropolitan University, 3-3-138 Sugimoto, Sumiyoshi-ku, Osaka, 558-8585 Japan; 3 National Institute for Environmental Studies, 16-2 Onogawa, Tsukuba, 305- 8506 Ibaraki Japan; 4 DeepForest Technologies Co., Ltd, 99 Tachiuri-Nakanocho, Shimogyo-ku, Kyoto, 600- 8006 Japan; 5 Biome Inc, 134 Chudoji-Minamimachi, Shimogyo-ku, Kyoto, 600-8813 Japan; 6 Tokyo University of Agriculture, 1-1-1 Sakuragaoka, Setagaya-ku, Tokyo, 156-8502 Japan; 7 Forestry and Forest Products Research Institute, 1 Matsunosato, Tsukuba, 300-1244 Ibaraki Japan; 8 Hokkaido University, North 8 West 5, Sapporo, 060-0808 Hokkaido Japan; 9 Faculty of Tropical Forestry, Universiti Malaysia Sabah, Kota Kinabalu, Sabah, 88400 Malaysia; 10 Forest Research Center, Mile 14, Sepilok Road, Sandakan, Sabah, 90007 Malaysia; 11 The Hakubi Center for Advanced Research, Kyoto University, Yoshida-Honmachi, Sakyo-ku, Kyoto, 606-8501 Japan

**Keywords:** Ecology, Ecology, Environmental sciences

## Abstract

Monitoring tropical ecosystem services such as carbon stock and biodiversity with satellite remote sensing is essential for addressing climate change and biodiversity loss, but collecting ground truth data is costly. We investigated whether Unmanned Aerial Vehicles (UAVs) can reduce the costs. First, we developed a method to estimate Above-Ground Carbon (AGC) and biodiversity index (mixing ratio of pioneer and late-successional species) based on data derived from UAV-RGB images in four Forest Management Units (FMUs) in Malaysia. Second, we tested whether adding UAV-based ground truth (i.e., estimated carbon and biodiversity index) improves satellite-based models. We built machine learning models to estimate AGC and biodiversity index based on Landsat metrics and inventory data across Malaysia and Indonesia (395 plots). Accuracy was low without local inventory data (287 plots outside the four FMUs; *R*^2^ = 0.43 and 0.46 for AGC and biodiversity, respectively). Adding UAV-based data (*n* = 934) significantly increased the accuracy (*R*^2^ = 0.51 and 0.48), which was comparable to the model with the full dataset including local inventory data (*R*^2^ = 0.53 and 0.60). These results underscore that integrating UAV and satellite analyses facilitates the monitoring of ecosystem services in tropical forests by reducing costs while maintaining accuracy.

**Supplementary Information:**

The online version contains supplementary material available at 10.1038/s41598-025-34938-9.

## Introduction

Tropical forests harbor high carbon stock and rich biodiversity^1–3^. However, deforestation and forest degradation are progressing^4–7^, which poses significant threats to tropical ecosystem services^5,8–11^. To halt the decline of ecosystem services and facilitate ecosystem recovery, there is growing global concern over the monitoring of ecosystem services across extensive tropical landscapes.

To cover the multiple dimensions of biodiversity, the concept of essential biodiversity variables (genetic composition, species populations, species traits, community composition, ecosystem structure, and ecosystem function) has been proposed^12^, among which tree community composition is an effective measure to evaluate tropical forest degradation^13^. Previous studies estimating forest carbon stock and tree community composition at a single Forest Management Unit (FMU) scale (from hundreds to thousands km²) have relied on regression models linking tree inventory data in a specific year and region with satellite images in the same year and region^14,15^. However, monitoring large areas across multiple FMUs over several years requires tree inventories at large spatial and temporal scales, which is a significant burden in terms of labor, money, and time. To enable large-scale forest monitoring, it is crucial to bridge the gap between fine-scale data from tree inventories and large-scale satellite data^16^.

In recent years, LiDAR and Hyperspectral Imaging (HSI) have been widely used in tropical forest assessments. LiDAR provides precise three-dimensional data for tree crown shapes and heights, aiding in Above-Ground Carbon (AGC) density estimation beyond traditional field plots^17,18^. LiDAR-derived laser penetration rates correlated with tree community composition across forests with various disturbance types in Borneo^19^. On the other hand, HSI captures spectral data reflecting plant chemistry, enabling species and trait diversity evaluation^17^. HSI-derived metrics such as Normalized Difference Vegetation Index (NDVI) correlate with tree species richness^16^.

While LiDAR and HSI are effective tools, both approaches require high financial costs and analytical expertise. On the other hand, UAVs equipped with RGB imaging have been used as a low-cost alternative to assess carbon stock in mangrove forests^20^, floristic biodiversity in beach-dominated temperate forest^21^, species distribution in subtropical China^22^, and to classify tree canopies in mixed forests in Japan^23^. The low-cost equipment and high spatiotemporal resolution provide further advantages to assess carbon stock and biodiversity in tropical forests, while having disadvantages such as limited payload, short flight duration, and restricted information acquisition (Table S1). Analyses on UAV-RGB images have been implemented in tropical regions to determine visible canopy characteristics such as canopy disturbance and flower phenology^24,25^, and to separate canopy trees from understory trees^24^. However, UAV-RGB images have been rarely used for carbon and biodiversity assessments in the tropical forests^26^ (Issue 1). This is likely because tropical forest is a challenging ecosystem due to the poor accessibility, dense tree canopies, and high tree species diversity.

There is another issue in ecosystem service monitoring at larger spatial scales using the UAV-derived information. Recently, accumulating and sharing data on tree inventory and biodiversity inside and across countries is being more common, which enhances ecosystem-service monitoring on the large spatial scale through analyzing them with satellite imagery. If carbon and biodiversity information can be obtained from UAV-RGB images and this information can be used with tree inventory networks as training dataset, costs for ground truthing will be significantly reduced. Although carbon stock could be mapped using airborne LiDAR sensors at a country scale^27,28^, we suggest the significance of tree inventory data for ecosystem service mapping because (1) airborne LiDAR is costly to apply to many countries, and (2) tree inventory data provide information on various aspects of biodiversity as well as carbon stock enabling the simultaneous evaluation of multiple ecosystem services^15^. There are so far no studies to test the utility of data from UAV-RGB images in the context of satellite-based forest monitoring using tree inventory network (Issue 2).

**Fig. 1:**
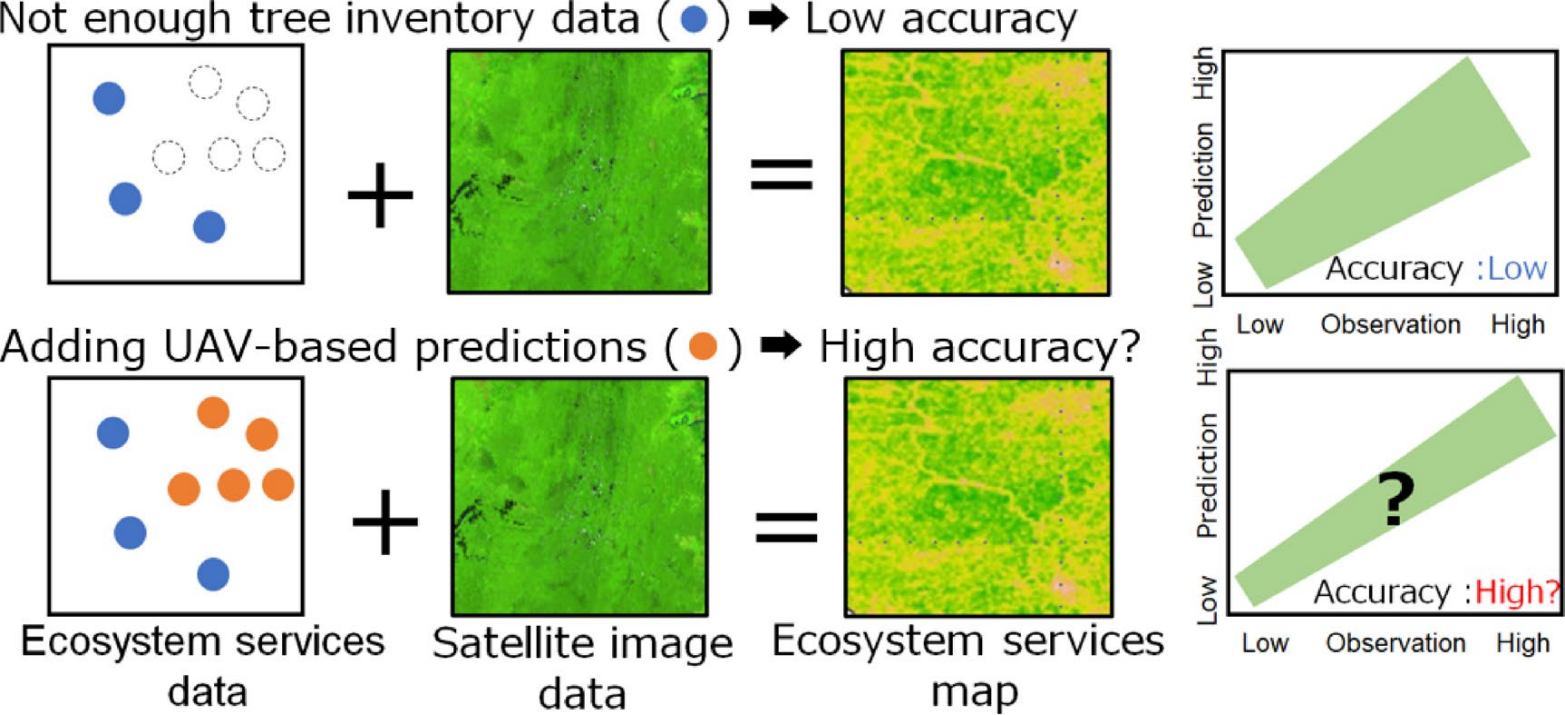
A diagram illustrating the research purpose. This study examines whether Unmanned Aerial Vehicles (UAVs) can reduce the costs for ground truthing required to estimate tropical ecosystem services such as Above-Ground Carbon (AGC) and biodiversity with satellite remote sensing. When tree inventory data are insufficient, the accuracy of models to predict ecosystem services is low (above). We hypothesize that inclusion of UAV-based information as ground truthing enhances model accuracy (below).

To address these issues, we developed a method to extract the information on carbon and biodiversity indicator using UAV-derived metrics, and examined whether incorporating UAV-based information with tree inventory data as ground truth improves the accuracy of models predicting the ecosystem-service metrics using satellite data (Fig. 1). This study involves three major steps. We developed a deep learning model to separate canopies of Dipterocarpaceae from canopies of other species in UAV-RGB images (step 1–1), and created a regression model to predict AGC and a biodiversity index using UAV-derived metrics (e.g., canopy structure, gap information, and dipterocarp crown area ratio from the deep learning model) (step 1–2). To develop the model for detecting dipterocarp canopy is important because Dipterocarpaceae dominate Bornean tropical forests^29^, exhibit high productivity^30^, and are key targets for harvesting. After those steps, we built a machine learning model to predict AGC and a biodiversity index, based on UAV-based information and tree inventory data, and satellite image information (step 2). In this study, we used the Functional Group (FG) ratio, defined as the mixing ratio of pioneer and late-successional species, as a biodiversity index.

## Results

I. Step 1–1 and 1–2: Identifying dipterocarp canopies and predicting AGC and FG ratio using UAV images

**Fig. 2:**
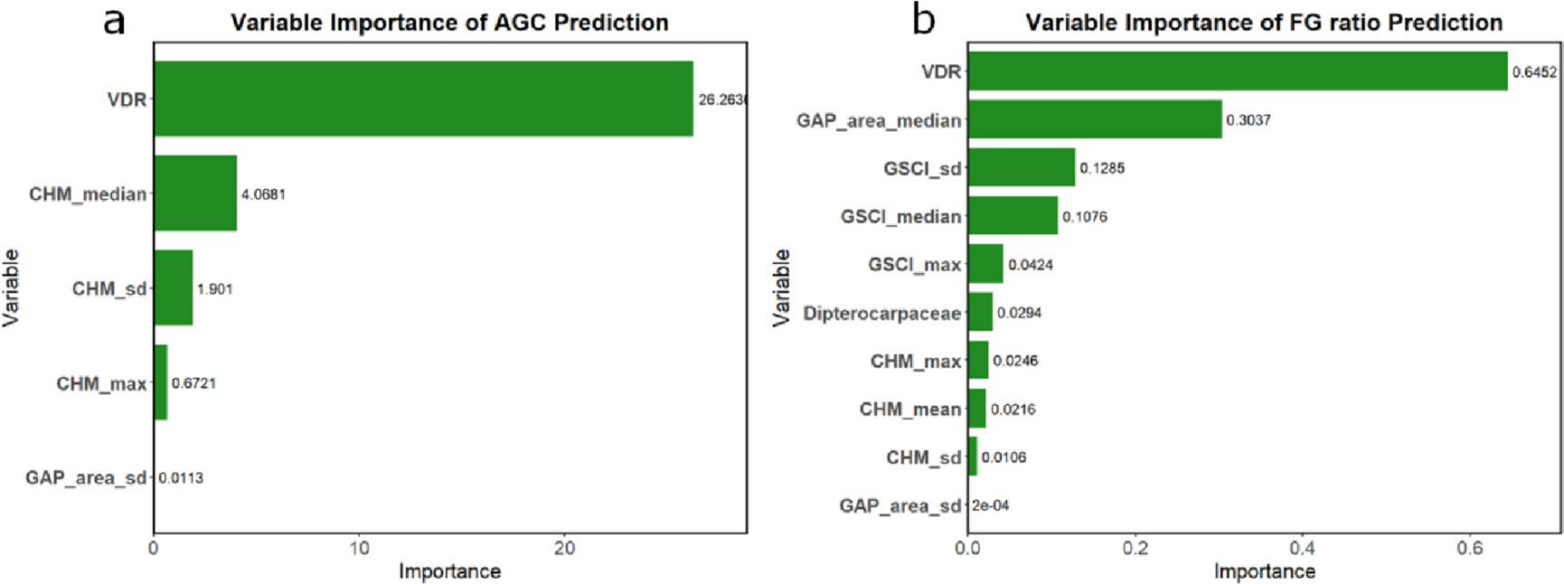
The variable importance of models to estimate (**a**) Above-Ground Carbon (AGC) and (**b**) biodiversity index (mixing ratio of pioneer and late-successional species) based on UAV-derived metrics such as canopy height and abundance of late-successional tree species. Vertical Distribution Ratio (VDR) represents the vertical distribution of vegetation inside the canopy. CHM (Canopy Height Model) reflects the height of the canopy. GAP_area indicates the area ratio of gaps in the plots. GSCI (Gap Shape Complexity Index) measures the complexity of gap shapes, and Dipterocarpaceae represents the area ratio of Dipterocarpaceae trees in the plots. “max” refers to the maximum value, while “sd” indicates standard deviation. The variables not shown in the graph have a coefficient of 0.

**Fig. 3:**
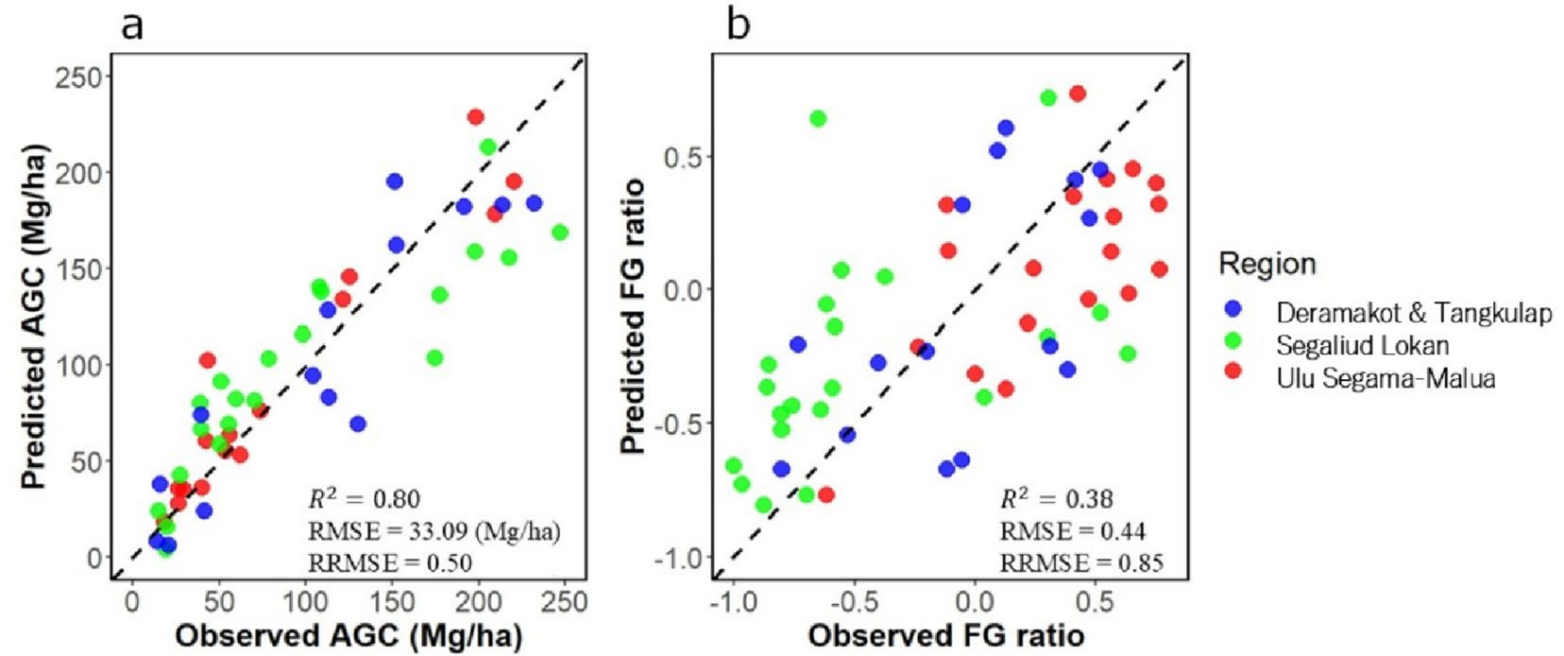
The relationship between observed and predicted values of Above-Ground Carbon (AGC, **a**), Functional Group (FG) ratio (**b**). Metrics derived from Unmanned Aerial Vehicle-RGB images were used for prediction. Each point represents a single plot, and the dashed line indicates the 1:1 line. Colors indicate forest management units: blue, Deramakot and Tangkulap; green, Segaliud Lokan; red, Ulu Segama-Malua. *R*^2^, Root Mean Square Error (RMSE) and Relative RMSE (RRMSE) are also shown.

The overall accuracy of the deep learning model to separate canopies of Dipterocarpaceae from canopies of other species in UAV-RGB images was 0.68. The user’s accuracy and producer’s accuracy are shown in Table 1. The relative importance of the variables in the models to predict AGC and FG ratio based on UAV-derived metrics is shown in Fig. 2. Vertical Distribution Ratio (VDR), the median, Standard Deviation (SD) and maximum of Canopy Height Model (CHM) and SD ratio of gap area to plot area were selected for the model to predict AGC, whereas VDR, the median and SD ratio of gap area to plot area, the SD, median and maximum of GSCI (Gap Shape Complexity Index), dipterocarp crown area ratio and the maximum, mean and SD of CHM were selected for the model to predict FG ratio (Fig. 2). *R*^2^ values were 0.80 and 0.38, Root Mean Square Error (RMSE) values were 33.09 Mg/ha and 0.44, and Relative RMSE (RRMSE; RMSE divided by the standard deviation of observed values) values were 0.50 and 0.85 for AGC and FG ratio, respectively. (Fig. 3). Above-Ground Carbon was underestimated in high-AGC plots and model residual was larger for the estimation of FG ratio for plots with high FG ratio (Fig. 3).

**Table 1:** The prediction accuracy of deep learning model to separate tree canopies between Dipterocarpaceae and other species.

		Predicted		
		Dipterocarpaceae	Other species	Producer’s accuracy
Observed	Dipterocarpaceae	60	25	0.71
	Other species	30	59	0.66
	User’s accuracy	0.67	0.70	0.68

II. Step 2: Predicting AGC and FG ratio based on satellite image information

**Fig. 4:**
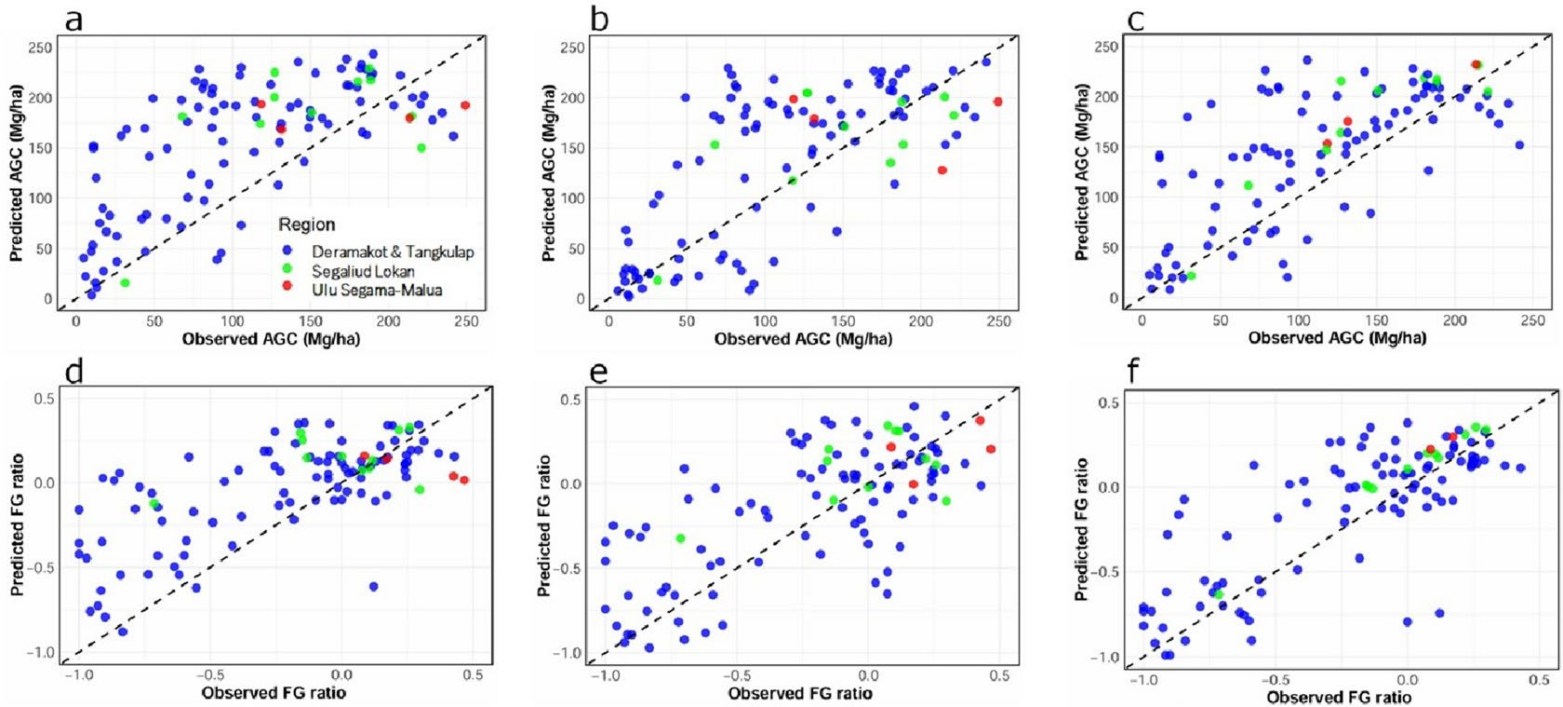
The relationships between observed and predicted Above-Ground Carbon (AGC) are shown in panels **a**, **b**, and **c**, and those for the Functional Group ratio (FG ratio) are shown in panels **d**, **e**, and **f**. Values were predicted by three types of machine learning models: Model 1 was established with tree inventory data from outside the target regions (**a** and **d**); Model 2 was established with local UAV-based information and tree inventory data outside the target regions (**b** and **e**); and Model 3 was established with tree inventory data from across Borneo, including the target regions (**c** and **f**). Each point indicates a single plot, and the dashed line indicates the 1:1 line. The different colors indicate forest management units (FMUs): blue, Deramakot and Tangkulap; green, Segaliud Lokan; and red, Ulu Segama-Malua FMU.

When the model with the tree inventory data outside the target FMUs was extrapolated to the target four FMUs (i.e., no local ground truthing), the model exhibited a low accuracy and significant biases (*R*^2^ = 0.43, RMSE = 70.60 Mg/ha, RRMSE = 1.00 for AGC, Fig. 4a; Table 2; *R*^2^ = 0.46, RMSE = 0.35, RRMSE = 0.92 for FG ratio, Fig. 4d; Table 2), while model with the local tree inventory data exhibited a greater accuracy and smaller bias (*R*^2^ = 0.53, RMSE = 58.18 Mg/ha, RRMSE = 0.82 or AGC, Fig. 4c; Table 2; *R*^2^ = 0.60, RMSE = 0.28, RRMSE = 0.76 for FG ratio, Fig. 4f; Table 2). On the other hand, the model accuracy was improved, and the model bias was mitigated when local UAV-based information was included as training data (*R*^2^ = 0.51, RMSE = 60.53 Mg/ha, RRMSE = 0.86 for AGC Fig. 4b; Table 2; *R*^2^ = 0.48, RMSE = 0.32, RRMSE = 0.85 for FG ratio, Fig. 4e; Table 2). For both AGC and FG ratio prediction in model 3, Shortwave Infrared 1 (SWIR1), SWIR2 and Green bands were the most important variables (Figure S2).

**Table 2:** The prediction accuracy of three types of machine learning models for Above-Ground carbon (AGC) and functional group (FG) ratio. *R*², root mean square error (RMSE) and relative RMSE (RRMSE) were calculated for 100 times and obtained mean and 95% confidence intervals (CIs) of *R*^2^, RMSE and RRMSE for each model. The “Source” column indicates the type of data used from the extrapolation regions to develop the machine learning models. “No additional data” means no ground-truth data from the target regions was used, “Plus unmanned aerial vehicle (UAV) data” means UAV-based AGC and FG ratio data (*n* = 934) was used, and “Plus tree inventory data” means ground-based AGC and FG ratio data (*n* = 107) was used.

Source	AGC			FG ratio		
	*R* ^2^	RMSE (Mg/ha)	RRMSE	*R* ^2^	RMSE	RRMSE
Model 1 No additional data	0.43 (CI: 0.39–0.47)	70.60 (CI: 68.05–73.55)	1.00 (CI: 0.96–1.04)	0.46 (CI: 0.42–0.50)	0.35 (CI: 0.33–0.36)	0.92 (CI: 0.88–0.96)
Model 2 Plus UAV data	0.51 (CI: 0.49–0.52)	60.53 (CI: 58.76–61.86)	0.86 (CI: 0.83–0.87)	0.48 (CI: 0.44–0.53)	0.32 (CI: 0.30–0.34)	0.85 (CI: 0.81–0.90)
Model 3 Plus tree inventory data	0.53 (CI: 0.49–0.56)	58.18 (CI: 55.72–60.55)	0.82 (CI: 0.79–0.86)	0.60 (CI: 0.53–0.65)	0.28 (CI: 0.26–0.31)	0.76 (CI: 0.71–0.84)

Prediction accuracy improved for both AGC and FG ratio with an increasing number of tree inventory data without UAV-based data (Figure S3.2) and an increasing number of UAV-based data without tree inventory data (Figure S3.4). However, the improvement in prediction accuracy saturated after incorporating more than 200 UAV-based data points.

## Discussion

Overall accuracy of the model was 68% in separating dipterocarp canopies from other species (Table 1), which was lower than the accuracy reported in previous studies on canopy classification using UAV-RGB imagery in temperate forests: overall accuracy to separate 4 to 14 classes, 82–89%^23,31,32^. This may be attributed to the high species diversity in tropical forests. Overlapping canopies of tropical forests could be another cause of errors because it complicates the delineation of individual tree canopies and reduce crown classification accuracy. We tried to mitigate this issue by over-segmentation, which divides each canopy into multiple polygons and ensures each polygon to contain single species^33^. However, in comparison to individual-based classification, the over-segmentation method may lead to the loss of important information, such as canopy shape, which could reduce classification accuracy.

To enhance the classification accuracy, integrating RGB images with additional data, such as multispectral data (e.g., Near Infrared (NIR), vegetation indexes), Digital Surface Model (DSM), CHM, or satellite-derived land-use history, is crucial^31,32,34,35^. We also highlight the need to separate Dipterocarpaceae into different functional groups or species to account for their intra-family variation. Some dipterocarp species (e.g., *Shorea leprosula*) grow fast and others (e.g., *Hopea nervosa*) grow slowly^36^. This ecological difference might affect their branch architecture and hence canopy structure^37^. The current classification of non-dipterocarp and dipterocarp species may be the oversimplification, reducing its accuracy and ecological significance. Given their wide distribution and ecological and economic importance, an improved model to identify dipterocarp canopies has the potential to contribute to forest monitoring and sustainable management across broader regions of Borneo.

We demonstrated that AGC was accurately predicted with UAV-RGB image information without biases across the four spatially remote FMUs (R² = 0.80) (Fig. 3a). This indicates the wide applicability of the model to lowland dipterocarp forests in Borneo. Furthermore, the model accuracy was comparable to the model to predict AGC in a mangrove ecosystem using UAV-RGB images (*R*^2^ = 0.81)^20^, and in Bornean tropical forests using LiDAR (R² = 0.81)^38^. This study demonstrates the potential of UAV-RGB imaging for AGC prediction in tropical forests with high biodiversity and dense canopies. Variables related to tree height, such as VDR and CHM, were identified as the most significant predictors of AGC (Fig. 2a), consistent with previous LiDAR-based studies in various climate zones (e.g., temperate region^39^; cool-temperate region^40^; boreal region^41^). Because UAV-RGB imaging was effective for AGC estimation even in tropical forests with dense canopies, UAV-RGB imaging might hold significant potential for carbon stock estimation across diverse ecosystems.

In comparison with AGC, the model accuracy was not high for the biodiversity indicator, i.e., FG ratio (R² = 0.38) (Fig. 3b). Using LiDAR-derived information, such as tree height data and laser penetration rates, one study evaluated the differences in tree community composition between forests with different magnitude of disturbance (R² = 0.71)^19^, which is strongly correlated with FG ratio^42^. Although RGB imagery cannot capture laser penetration rates, it can provide canopy structure information such as VDR (vertical canopy structure), GSCI (gap shape complexity), and CHM (canopy height), which were important predictors of FG ratio in this study (Fig. 2b). Previous research has shown that VDR is useful for predicting forest diversity (e.g., species richness)^21^, GSCI for understory diversity (e.g., species richness)^22^, and CHM for classifying tropical forest canopies^35^. While RGB imaging provides valuable information, it offers fewer data types compared to LiDAR or hyperspectral imaging making it challenging to improve FG ratio prediction accuracy. Enhancing the canopy classification model (Step 1–1) might lead to a more accurate FG ratio estimation.

The model to predict AGC and FG ratio with satellite image data and local tree inventory data inside the target FMUs showed greater accuracy than the model using only tree inventory data outside the target FMUs (Table 2; Δ*R*² = 0.10 and ΔRRMSE = 0.18, Fig. 4a vs. 4c for AGC; Δ*R*² = 0.14 and ΔRRMSE = 0.16, 4d vs. 4f for FG ratio). This result demonstrates the significance of local tree inventory data for maintaining prediction accuracy, and the significance of extensive ground truthing for large-scale ecosystem service monitoring. However, establishing plots and identifying species are labor-intensive and costly particularly in diverse tropical forests, presenting significant challenges for large-scale and continuous forest monitoring. For AGC prediction, advanced technologies such as Airborne-LiDAR can estimate carbon stock with minimal tree inventories despite their broad evaluation range^27,28^. However, their high costs and the need for specialized expertise render them impractical for applications across tropical regions. Therefore, the implementation of UAV-RGB imaging is essential to address these challenges.

Adding local UAV-based information to the training dataset significantly improved the accuracy of the model that relied solely on tree inventory data outside the target regions for both AGC (Table 2; Δ*R*² = 0.08 and ΔRRMSE = 0.14, Fig. 4a vs. b) and FG ratio (Table 2; Δ*R*² = 0.02 and ΔRRMSE = 0.07, Fig. 4d vs. e). For AGC, the model with local UAV-based data was comparable to the one with local tree inventory data (Fig. 4b vs. 4c; Table 2). In contrast, for FG ratio, the accuracy was not comparable while adding local UAV-based data significantly mitigated the model bias (Fig. 4e vs. 4f; Table 2). Model accuracy of FG ratio prediction with 90% of the local UAV-based data was *R*^2^ = 0.492 (Figure S3.4), which corresponds to the model accuracy with 10% of local tree inventory data (*n* = 7–8: *R*^2^ = 0.492) (Figure S3.2). Note that the models developed in this study might have limited applicability to diverse vegetation types in the tropics because our training data were in lowland dipterocarp forests that are abundant in Southeast Asia. However, our approach combining UAV and satellite has a potential to apply to forests outside dipterocarp forests and we suggest that this has a strong potential for improving cost-effective tropical forest monitoring, particularly where tree inventory data are scarce.

Unmanned aerial vehicles have been used to estimate carbon stock and biodiversity in limited areas (tens and hundreds ha)^16,20,35^. However, UAVs may help assess carbon stock and biodiversity over larger areas by integrating data of tree inventory network on a broader scale, such as National Forest Inventories (NFIs). Large-scale tree inventories are costly and challenging to conduct frequently across all plots^43^. Therefore, low-cost UAVs with high temporal resolution is complimentary and useful for sustainable forest monitoring. Interestingly, adding UAV-based FG ratio data improved the satellite-based model despite the low accuracy of FG ratio prediction using UAV-derived metrics (Step 1–2). This might be because nearly 1000 data addition of UAV-based FG ratio help the machine-learning process. Creating a large number of training dataset might be another advantage of combining UAV analyses with tree inventory network.

In this study, we have tested the utility of UAV-RGB image analyses across multiple regions varying management history, suggesting that combined analyses of UAV-based information and tree inventory data is useful across Bornean tropical forests. Furthermore, although this study focused primarily on tropical forests—due to the challenges posed by their high tree species diversity and dense canopies—this approach may be applicable to forests in other climate zones. The integration of advanced technologies such as LiDAR and HSI could further improve the effectiveness of UAV analyses as an alternative of tree inventories. We suggest that this approach reduces the reliance on labor-intensive and expensive tree inventories, making ecosystem-service monitoring feasible, particularly for regions with limited resources. By demonstrating the utility of UAV-RGB analyses in large-scale forest monitoring, this study paves the way for scalable solutions for advance climate action and biodiversity conservation.

## Materials and methods

I. Tree inventory and UAV survey

**Fig. 5:**
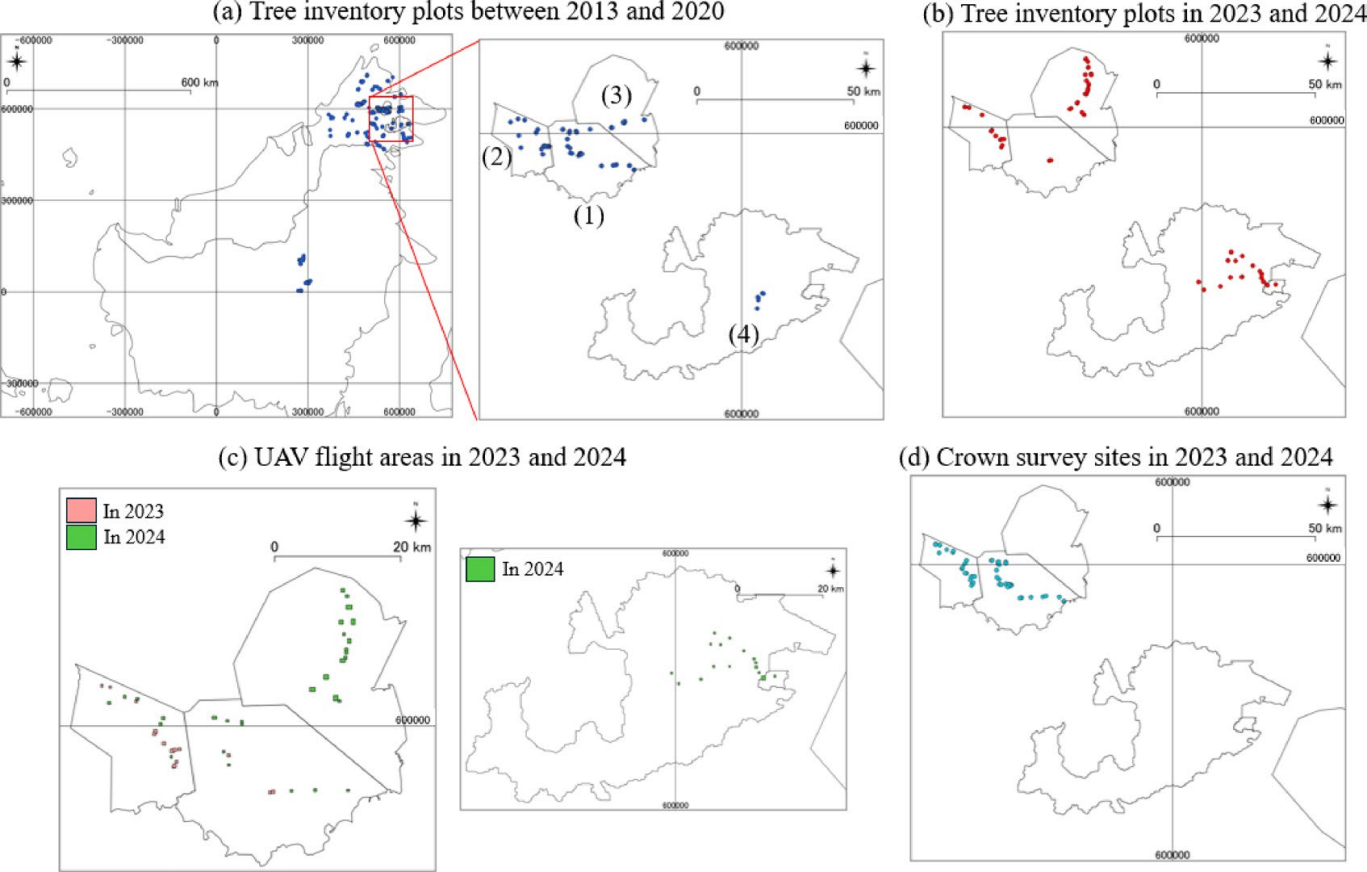
The locations of tree inventory plots used in Step 2 (*n* = 545) are shown in the left panel of Fig. 5a. The right panel of Fig. 5a shows four Forest Management Units (FMUs) including (1) Deramakot, (2) Tangkulap, (3) Segaliud Lokan, (4) Ulu Segama–Malua. The number of plots for each FMU is 100 in Deramakot and Tangkulap, 10 in Segaliud Lokan, and 6 in Ulu Segama–Malua. The locations of tree inventory plots used in Steps 1–2 are presented in Fig. 5b. The number of plots is 16 in Deramakot and Tangkulap, 23 in Segaliud Lokan, and 20 in Ulu Segama–Malua. Figure 5c shows the areas of UAV‑RGB images captured in 2023 and 2024, which are indicated by pink and green colors, respectively (*n* = 14 and 48, respectively). Figure 5d shows the locations of plots where crown surveys were conducted, which are indicated with blue color (*n* = 57). Crown surveys were not conducted for all plots. Horizontal and vertical grid lines indicate latitude and longitude in WGS 84 / UTM zone 50 N. These images were generated using QGIS 3.18 (QGIS Development Team, USA, https://qgis.org/).

We collected the data of tree inventory at 59 plots in four FMUs including Deramakot, Tangkulap, Segaliud Lokan and Ulu Segama-Malua FMU (established in 2023 and 2024) to develop the UAV-based model (Step 1–2) and at 545 plots distributed across Sabah, Malaysia, and East Kalimantan, Indonesia (established between 2013 and 2020) to develop the satellite-based model (Step 2) (Fig. 5a, b). These plots were mostly circular with a 20‑m radius, although some were square. All plots were established in lowland dipterocarp forests where legal logging was being conducted for timber production (see Supplementary 4 for details on site and plot information). Trees with a diameter at breast height (DBH) of 10-cm or greater were recorded for their DBH and identified to at least genus level. Global Positioning System (GPS) coordinates were collected with Garmin portable GPSs by averaging for two hours. Above-Ground Carbon stock of each tree was estimated using the allometric equation ^44^ (See Supplementary 5 for details on calculation methods).

We also calculated FG ratio by assigning scores to trees based on functional groups (pioneer genera and late-successional genera)^42^, field guides, and taxonomists’ perspectives (pioneer genera = − 1; late-successional genera = 1; others = 0), summing the scores for all trees, and dividing it by the number of all trees^42^. We integrated a large dataset and species identification was conducted by local experts who have different levels of identification skill. Therefore, it was important to use a simple indicator such as FG ratio to minimize observer-related inconsistencies. On the other hand, the FG ratio is a single-dimensional proxy of biodiversity and does not account for richness, evenness, or phylogenetic diversity. This limitation should be considered when interpreting the results.

Using a commercially available UAV (Mavic 2 Pro with an RGB Hasselblad L1D-20c sensor), we captured over 500-ha of UAV-RGB imagery in June and October 2023 and September 2024 in Deramakot and Tangkulap, in June 2024 in Segaliud Lokan, and in August 2024 in Ulu Segama-Malua. We generated orthomosaic photos and CHMs (Fig. 5c; see Supplementary 6 for flight and processing details).

II. Step 1–1: Identifying dipterocarp crowns in UAV-RGB images

To estimate carbon stock and the biodiversity index based on UAV-RGB images, we first developed a deep learning model to identify dipterocarp crowns in UAV-RGB imagery. Dipterocarpaceae dominate forest canopy of old growth forests in Southeast Asia (e.g., their average abundance based on tree number in Borneo is 21.9%^29^), and their proportion in a stand is closely related to forest carbon stock and biodiversity^13,42^. Therefore, we expect that canopy coverage of Dipterocarpaceae may aid in explaining carbon stock and biodiversity.

We identified individual dipterocarp canopies in UAV-RGB imagery inside the vegetation plots and their surrounding forest in two FMUs (Deramakot and Tangkulap) in June and October 2023 and September 2024 (Fig. 5d, Table S7; See Supplementary 8 for detail of dipterocarp crown delineation and training data preparation from UAV-RGB imagery).

Canopy polygon image data were used as training and test data to develop a model for separating dipterocarp and non-dipterocarp canopies using UAV-RGB imagery (Fig. 5c; Table S7; See Supplementary 9 for details on UAV-based canopy segmentation and training dataset preparation). We employed deep learning model based on Convolutional Neural Network (CNN) with EfficientNet-B4^45^ in DF Scanner Pro (DeepForest Technologies Co., Ltd., Japan, https://deepforest-tech.co.jp/) (Figure S10, Step 1–1). The model was trained using a batch size of 16, a learning rate of 0.01, and for a total of 30 epochs. We then extrapolated the deep learning model to the entire segmented-UAV images and obtained the class information, “Dipterocarpaceae” or “Others”, across the entire UAV-RGB images in DF Scanner Pro (Fig. 5c, Table S7, Figure S8).

III. Step 1–2: Predicting AGC and FG ratio based on UAV-derived metrics

Using the deep learning model to estimate dipterocarp crown area and other metric based on UAV-RGB images acquired in the same year as plot establishment, we developed regression models to assess AGC and FG ratio of plots in four FMUs including Deramakot, Tangkulap, Segaliud Lokan and Ulu Segama-Malua (Fig. 5b, c, d; Table S7). All analyses below were conducted using R 4.1.1^46^. We obtained three types of UAV-derived metrics for each plot; (1) variables related to canopy height, (2) variables related to gaps and (3) variables related to canopy class (Dipterocarpaceae or other species) (see Supplementary 11 for details on how these variables were calculated).

We excluded three plots with AGC greater than 250 Mg/ha from 59 plots data in 2023 and 2024 (Table S7), because most plots were only 0.125 ha, where a single exceptionally large tree could inflate per-hectare AGC, resulting in unrealistically high values. Then, we standardized the explanatory variables and applied them to the Least Absolute Shrinkage and Selection Operator (LASSO) regression using the glmnet package in R for variable selection^47^. Then, we developed the linear regression model between AGC and selected variables (Figure S10, Step 1–2). As for FG ratio, we employed a logistic regression model to predict values between 0 and 1 (Figure S10, Step 1–2). To apply the logistic regression model, FG ratio was transformed by adding 1 to the original FG ratios and dividing by 2. Leave-One-Out Cross-Validation was used to assess the model accuracy. Model accuracy was assessed using *R*^2^, RMSE and RRMSE, based on the standard deviation of observed values, were used to assess model accuracy. We extrapolated the regression models to the entire UAV-RGB images to obtain training dataset (AGC and FG ratio) for satellite analyses described below. First, we randomly created twenty circular buffers with a radius of 20-m in forested areas across forty-eight UAV-RGB images captured in 2024 (each approximately 10 ha) across four FMUs (Fig. 5c, Table S7, Figure S12) including Deramakot, Tangkulap, Segaliud Lokan, and Ulu Segama-Malua. The total number of buffers was 340 in Deramakot-Tangkulap, and 300 in Segaliud Lokan and 320 in Ulu Segama-Malua, respectively. Then we obtained the variables related to tree height, canopy gaps and canopy classes, which were used as explanatory variables in the models. We extrapolated the linear and logistic regression models to the buffers to predict AGC and FG ratio. As for FG ratio, the predicted values have a range of 0 to 1, so we re-transformed them to the range of − 1 to 1 by multiplying by 2 and subtracting 1. We removed the buffers with greater than 250 AGC Mg/ha, yielding UAV-derived AGC and FG ratio data at 941 locations.

IV. Step 2: Predicting AGC and FG ratio based on satellite image information

To predict AGC and FG ratio in the four FMUs (Deramakot, Tangkulap, Segaliud Lokan, and Ulu Segama-Malua), we used the information of tree inventory across multiple regions in Borneo (Sabah, Malaysia, and East Kalimantan, Indonesia) established from 2013 to 2020 (Fig. 5a; *n* = 545). Plots with greater than 250 Mg/ha AGC (*n* = 151) were removed.

We utilized Landsat metrics and Landsat-derived disturbance history (See Supplementary 13 for details on satellite image preprocessing). The Landsat metrics include the surface reflectance values of seven bands (Aerosol (Aero), Red, Green, Blue, SWIR1, SWIR2, and NIR), as well as NDVI (Equation S14.1), Normalized Difference Water Index (NDWI; Equation S14.2), Normalized Difference Soil Index (NDSI; Equation S14.3) and Enhanced Vegetation Index (EVI; Equation S14.4). The mean values of 15 randomly sampled points within each plot were used as representative values for each Landsat metric.

To derive disturbance history of each plot, we analyzed the time-series changes in Normalized Burn Ratio (NBR; Equation S14.5) for each pixel from 1980 to the year of plot establishment using Landsat-based detection of trends in disturbance and recovery (LandTrendr) tool of Google Earth Engine (GEE)^48^ and calculated the following three indices within 20-m radius plots (See Supplementary 15 for details on disturbance detection and NBR-based indices calculation using LandTrendr).

### Yod

The most recent year in which a disturbance occurred.

### Mag

The amount of change in NBR inside a pixel due to the most recent disturbance.

### Rate

The recovery speed of NBR form the most recent disturbance to recovery period.

A machine learning model with the Random Forest^49^ algorithm was developed to estimate AGC and FG ratio with satellite image information (Figure S10, Step 2), using the caret package in R^50^, with the mtry parameter tuned across values from 1 to 5, in four target regions: Deramakot and Tangkulap FMU (*n* = 92), Segaliud Lokan (*n* = 11), and Ulu Segama-Malua FMU (*n* = 4) using the information of the plots established across Borneo (Fig. 5a; See Supplementary 16 for details on random forest predictions adjustment). An overall goal of this study is to test if the inclusion of UAV-based information improves model accuracy for the plot in the target regions. For this purpose, we developed the models with three different training datasets. The training data of model 1 includes only tree inventory data outside the target regions (*n* = 287), representing the model performance in the situation where no local ground truth data is available. On the other hand, the training data of model 2 and 3 includes tree inventory data outside the target regions (*n* = 107) plus UAV-based information (*n* = 934; derived in step 1–2) and tree inventory data insides the target region, respectively. They represent the model performances with the ground truth information of local UAV survey data and local tree inventory data, respectively. Prediction accuracy of *R*^2^, RMSE and RRMSE for each model was assessed using the test data with the cross-validation approach (i.e., plots inside the target regions that were not included in the training dataset; see Figure S17).

## Supplementary Information

Below is the link to the electronic supplementary material.

Supplementary Material 1

## Data Availability

The datasets used and analysed during the current study are available from the corresponding author on reasonable request.
