# Supplementary material for: Quantifying carbon stock and tree community composition in tropical forests through combining satellite and UAV analyses
Source: Sci Rep. 2026 Jan 23;16:5442. doi: 10.1038/s41598-025-34938-9 (PMC12886868; doi:10.1038/s41598-025-34938-9)
Supplement: Supplementary file 1 — Supplementary Material 1 [file 41598_2025_34938_MOESM1_ESM.docx]

Supplementary 1: Advantages and disadvantages of using Unmanned Aerial Vehicle (UAV)-RGB imaging

Table S1

Summary of the main advantages and disadvantages of UAV‑based RGB imaging compared with alternative remote‑sensing methods such as Light Detection and Ranging (LiDAR) and Hyper Spectral Imaging (HSI).

| Advantages |  |
| --- | --- |
| Low cost | UAV-RGB imaging is a cost-effective solution for data collection compared to LiDAR or HSI [1, 2]. |
| Aerial data collecting | UAVs are particularly advantageous in high-risk or hard-to-access areas compared to tree inventories [3], making them especially useful in tropical regions. |
| High spatial resolution | UAVs can fly close to the tree canopy and provide high spatial resolution data compared to aircrafts or satellites [1, 4]. High spatial resolution is required to monitor heterogeneous forests, such as tropical forests [5]. |
| High temporal resolution | UAVs offer high temporal resolution because their ability to quickly collect data enables repeated surveys inside a short period compared to tree inventories [6, 7]. |
| Ease of interpretation | RGB imagery is straightforward to interpret, and annotate compared to LiDAR or HSI [8]. |
| Disadvantages |  |
| Limited payload & flight duration | UAVs can carry only small equipment and have short flight times compared to aircrafts [1]. |
| Weather sensitivity | UAVs are affected by atmospheric conditions like fog, heavy rain, and winds compared to aircrafts [1]. |
| Limited to visual data | RGB imaging captures only the visible spectrum, lacking the ability to provide important spectral information about a specimen's chemical and physical properties compared to HSI [9]. |
| Sensitivity to sun light | RGB imagery is highly sensitive to environmental factors such as sun-light conditions, and time of the day, which can lead to variations in image quality compared to LiDAR [10]. |

**Supplementary 2: Calculation methods of variable importance**

The variable importance was calculated using Mean Decrease in Gini (MDG), which is based on Gini Impurity. The importance in the models for a) Above-Ground Carbon (AGC) prediction and b) Functional Group (FG) ratio prediction. The variables used for the machine learning model are shown in the graphs. We utilized 7 bands of surface reflectance rate from Landsat 8 and 9: Aerosol (Aero), Red, Green, Blue, Shortwave Infrared 1 (SWIR1), SWIR2, and Near InfraRed (NIR). Additionally, we calculated the Normalized Difference Vegetation Index (NDVI), Normalized Difference Water Index (NDWI), and Normalized Difference Soil Index (NDSI) from the surface reflectance values. We also incorporated information on forest disturbance history, including yod, which refers to the most recent year in which a disturbance occurred, mag, which represents the most recent disturbance magnitude, and rate, which measures the rate of change in the affected area during the most recent disturbance.


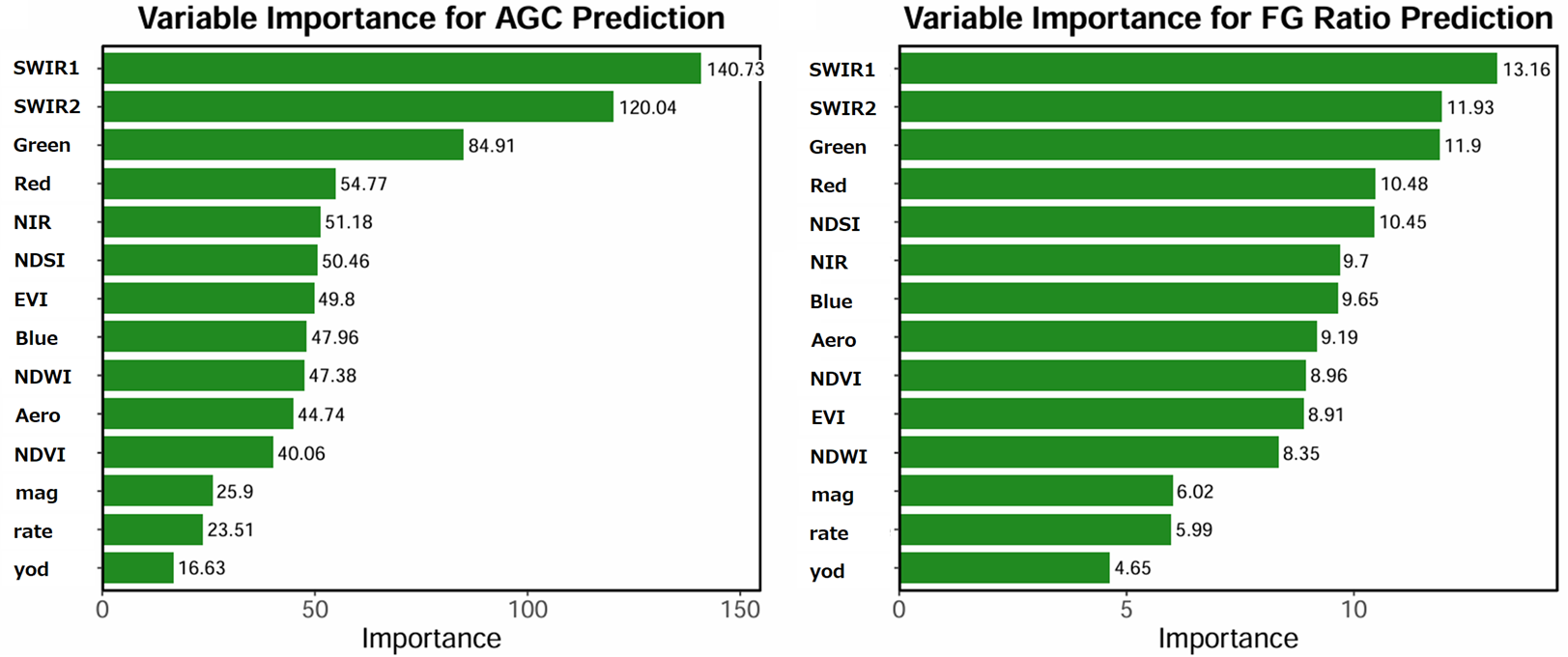


**Figure S2**

We calculated the variable importance using the full model of model 3 each for AGC prediction and FG ratio prediction. The variable importance was calculated using Mean Decrease in Gini (MDG), which is based on Gini Impurity.

**Supplementary 3: Additional analyses for model accuracy and data availability**


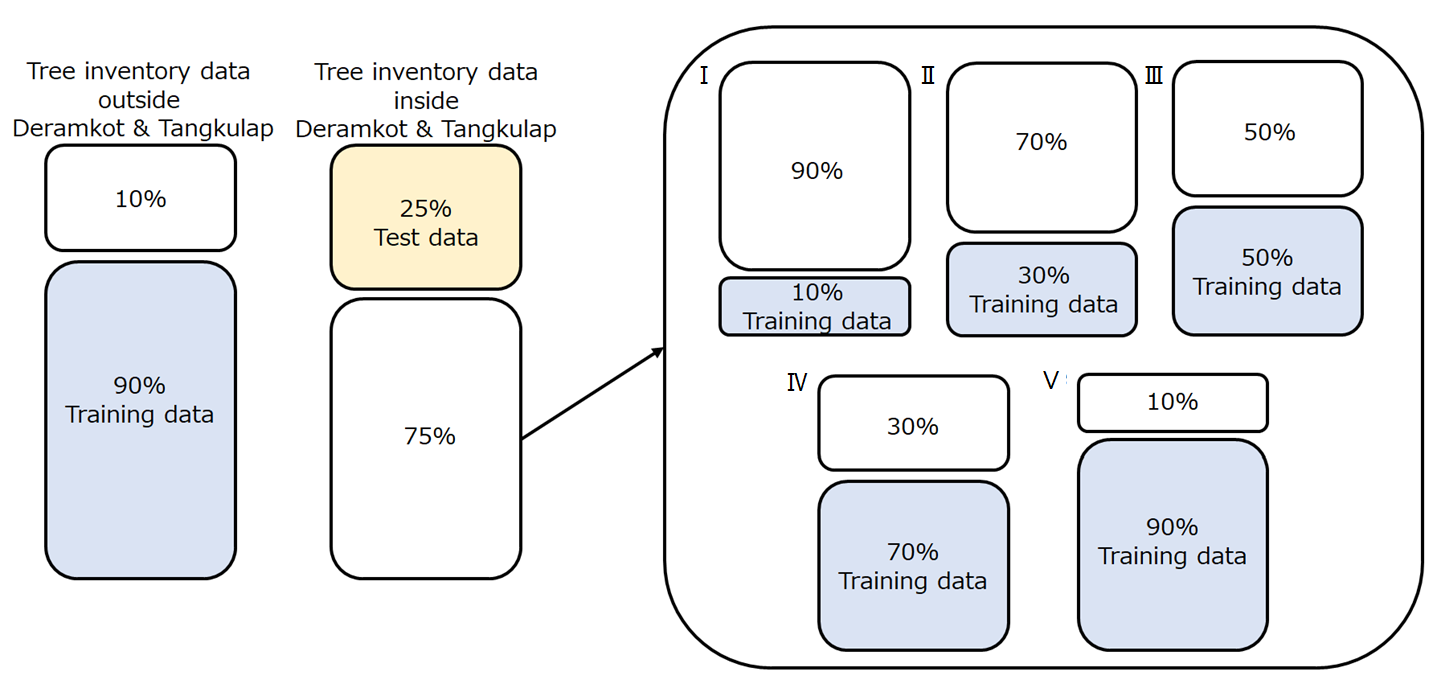


Figure S3.1

We examined how the number of tree inventory data inside a forest management unit (FMU) affect prediction accuracy of Above-Ground Carbon (AGC) and Functional Group (FG) ratio in the FMU by developing five models incorporating different number of tree inventory data. For this analysis we used the data in Deramakot and Tangkulap FMUs where many inventory data are available (n = 92). For these models, 25% of the tree inventory data inside the FMU was used as test data. The remaining 75% of the tree inventory data was used for training, but we include different number of training data in the models: 10% of remaining data (model Ⅰ, 30% (model Ⅱ), 50% (model Ⅲ), 70% (model Ⅳ), and 90% (model Ⅴ) (n = 7–8; n = 23–24; n = 39–41; n = 54–57; n = 70–73, respectively), and combined them with 90% of the tree inventory data from outside the FMUs. We conducted 4-fold cross-validation, ensuring that all tree inventory data from inside the FMUs was used as test data once. *R*², Root Mean Square Error (RMSE) and Relative RMSE (RRMSE) were calculated based on predicted and observed values, with the entire process repeated 50 times to compute the mean and 95% Confidence Intervals (CIs) for each model. In the figure, blue and orange represent the training and test data, respectively.


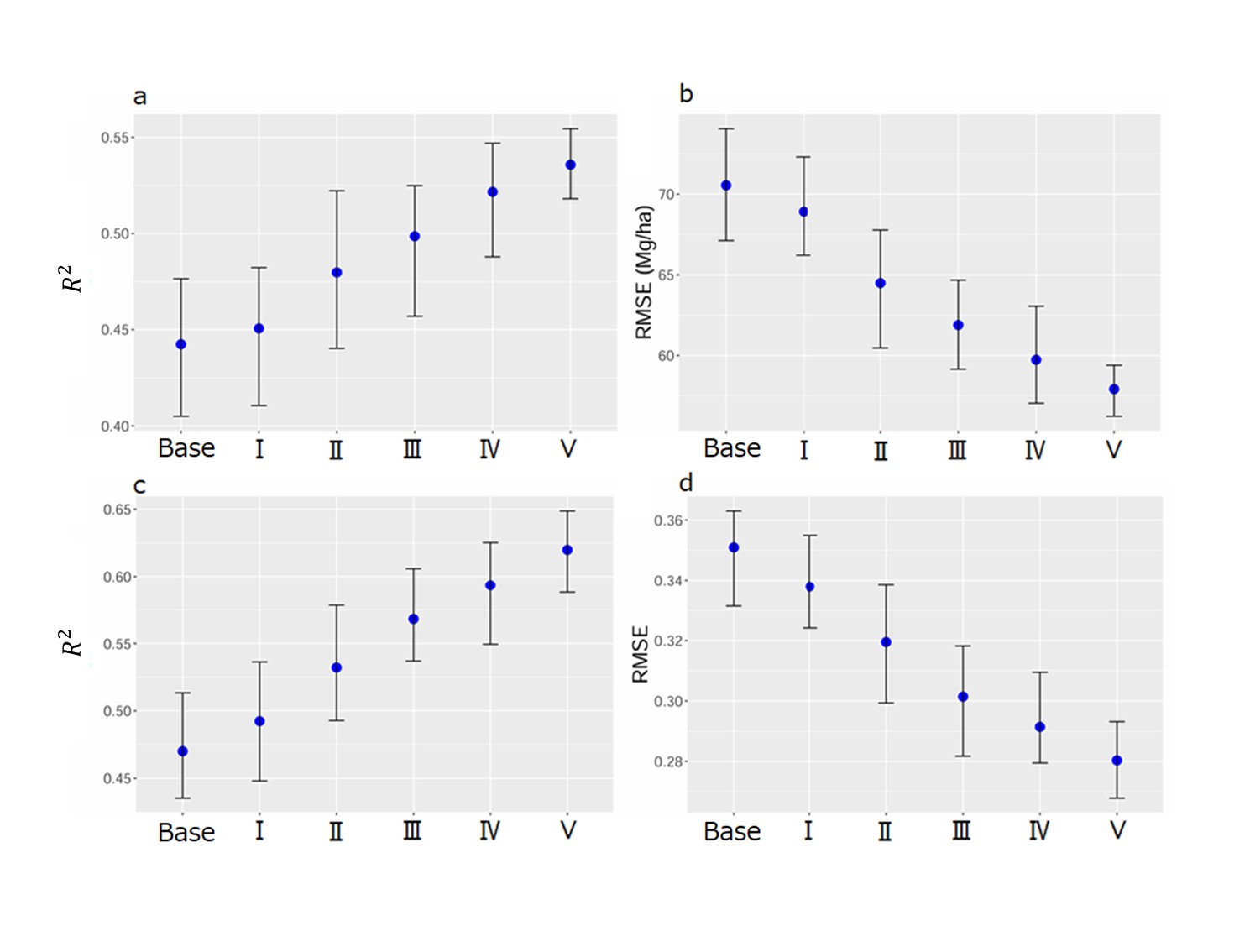


| Source | AGC | | | FG ratio | | |
| --- | --- | --- | --- | --- | --- | --- |
|  | *R*^2^ | RMSE (Mg/ha) | RRMSE | *R*^2^ | RMSE | RRMSE |
| Base. No additional data | 0.44  (CI: 0.41–0.48) | 70.55  (CI: 67.12–74.05) | 1.00  (CI: 0.95–1.05) | 0.47  (CI: 0.44–0.51) | 0.35  (CI: 0.33–0.36) | 0.94  (CI: 0.89–0.97) |
| Ⅰ. Plus tree inventory data (10%) | 0.45  (CI: 0.41–0.48) | 68.91  (CI: 66.21–  72.3) | 0.97  (CI: 0.94–1.02) | 0.49  (CI: 0.45–0.54) | 0.34  (CI: 0.32–0.36) | 0.90  (CI: 0.87–0.95) |
| Ⅱ. Plus tree inventory data (30%) | 0.48  (CI: 0.44–0.52) | 64.48  (CI: 60.46–67.77) | 0.91  (CI: 0.85–0.96) | 0.53  (CI: 0.49–0.58) | 0.32  (CI: 0.30–0.34) | 0.85  (CI: 0.80–0.90) |
| Ⅲ. Plus tree inventory data (50%) | 0.50  (CI: 0.46–0.53) | 61.88  (CI: 59.15–64.67) | 0.87  (CI: 0.84–0.91) | 0.57  (CI: 0.54–0.61) | 0.30  (CI: 0.28–0.32) | 0.81  (CI: 0.75–0.85) |
| Ⅳ. Plus tree inventory data (70%) | 0.52  (CI: 0.49–0.55) | 59.73  (CI: 57.03–63.05) | 0.84  (CI: 0.81–0.89) | 0.59  (CI: 0.55–0.63) | 0.29  (CI: 0.28–0.31) | 0.78  (CI: 0.75–0.83) |
| Ⅴ. Plus tree inventory data (90%) | 0.54  (CI: 0.52–0.55) | 57.91  (CI: 56.22–59.39) | 0.82  (CI: 0.79–0.84) | 0.62  (CI: 0.59–0.65) | 0.28  (CI: 0.27–0.29) | 0.75  (CI: 0.72–0.78) |

**Figure S3.2**

We compared six models with different numbers of training data inside the target forest management unit (FMU) to examine the impact of the number of tree inventory data on the accuracy of predicting Above-Ground Carbon (AGC) and Functional Group (FG) ratio. Materials and methods are described in Figure S3.1. *R*², RMSE and RRMSE are shown in graphs a and b for AGC, respectively, and in graphs c and d for FG ratio, respectively. First model was constructed using only tree inventory data outside the FMUs. The Second to Sixth models progressively included 10%, 30%, 50%, 70%, and 90% of tree inventory data from inside the FMUs, excluding test data, along with 90% of the data from outside the FMUs. We found that the prediction accuracy of both AGC and FG ratio improves with increasing the number of tree inventory data inside the target regions.


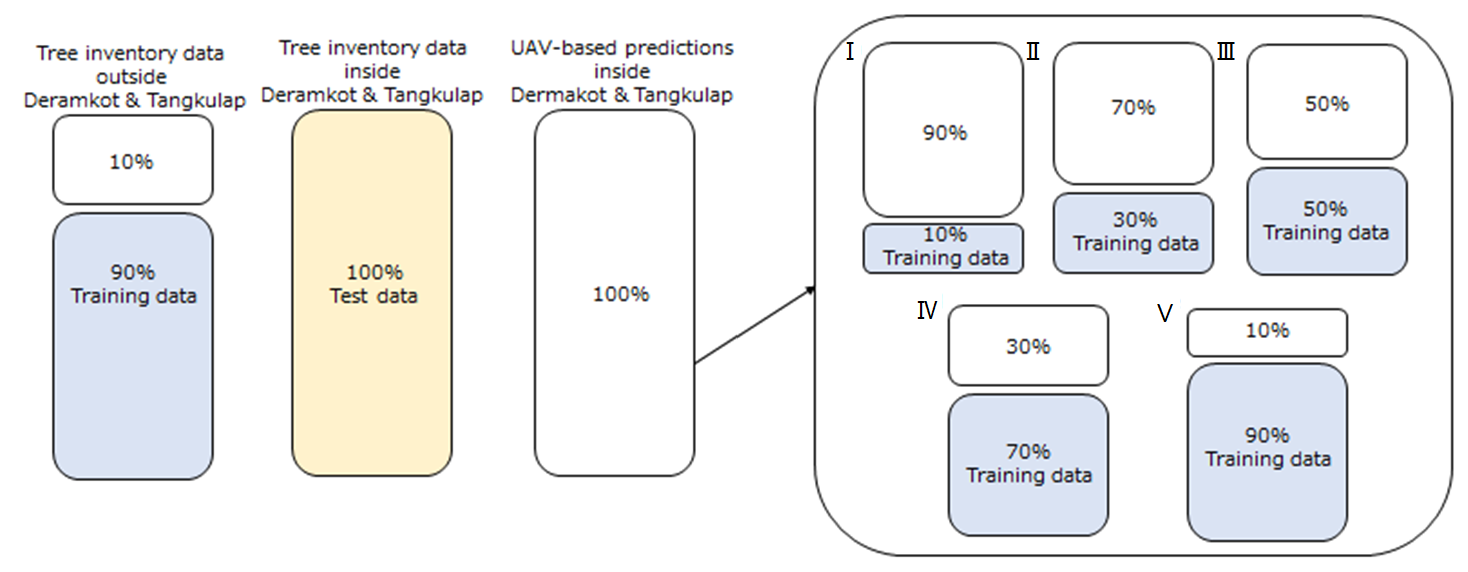

**Figure S3.3**

We examined how the number of Unmanned Aerial Vehicle (UAV)-based information inside a forest management unit (FMU) affect prediction accuracy of Above-Ground Carbon (AGC) and Functional Group (FG) ratio in the FMU by developing five models incorporating different number of UAV-based information. For this analysis we used the data in Deramakot and Tangkulap FMUs where many inventory data are available (n = 92). 90% of tree inventory data outside the target FMUs were randomly selected for model establishment. Different number of UAV-based information inside the FMUs were also included as training data: 10% (Ⅰ) 30% (Ⅱ), 50% (Ⅲ), 70% (Ⅳ), and 90% (Ⅴ) (n = 33; n = 100; n = 167; n = 233; n = 300). Under each condition, we established regression models to predict AGC and FG ratio based on UAV RGB data using subsets (25%) of all plot data and removed the plots used to established the models from test data so that no data were used simultaneously for both training the regression model in Step 1-2 and testing. *R*², Root Mean Square Error (RMSE) and Relative RMSE (RRMSE) were calculated based on predicted and observed values, with the entire process repeated 50 times to compute the mean and 95% Confidence Intervals (CIs) for each model. In the figure, blue and orange represent the training and test data, respectively.

**
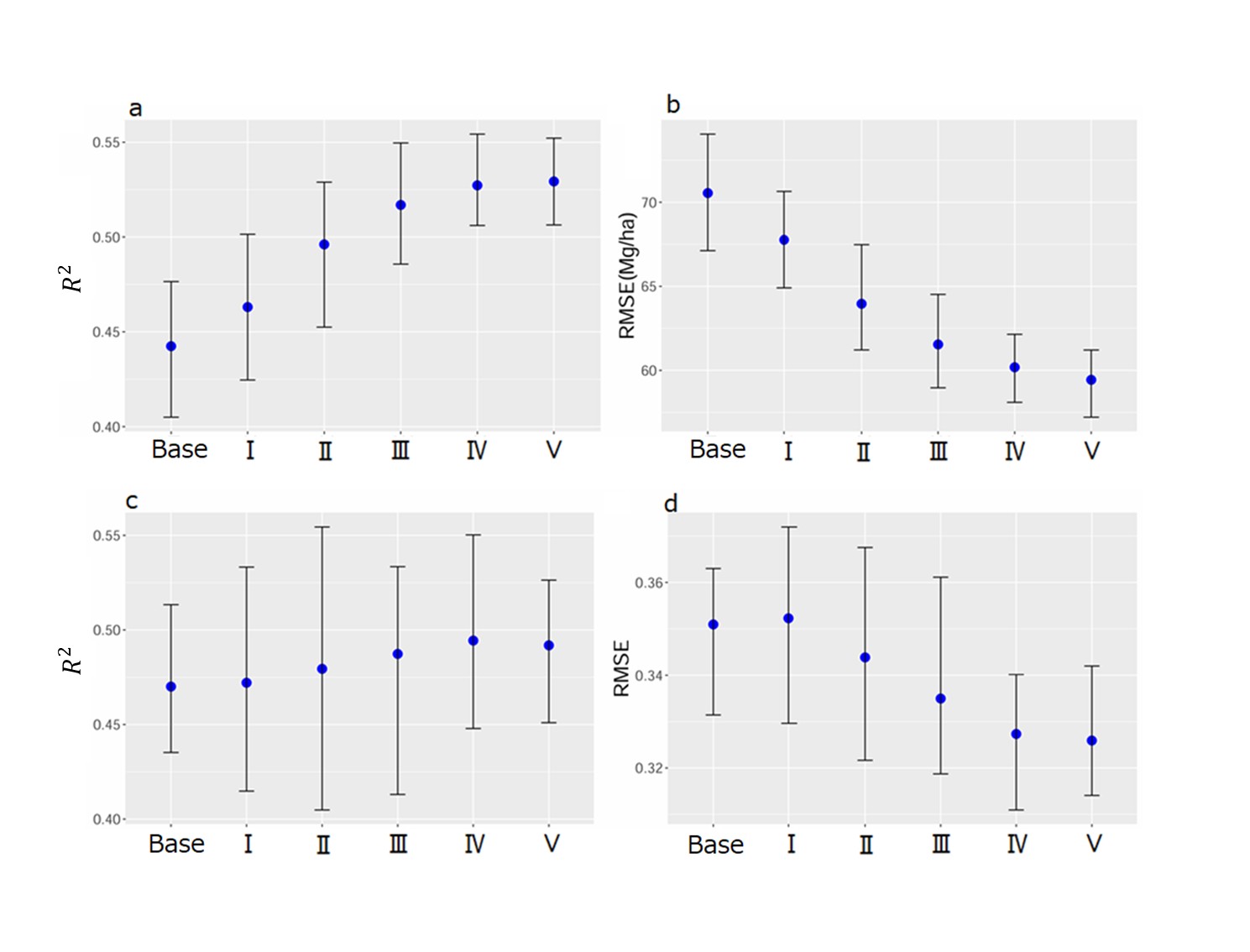
**

| Source | AGC | | | FG ratio | | |
| --- | --- | --- | --- | --- | --- | --- |
|  | *R*^2^ | RMSE (Mg/ha) | RRMSE | *R*^2^ | RMSE | RRMSE |
| Base. No additional data | 0.44  (CI: 0.41–0.48) | 70.55  (CI: 67.12–  74.05) | 1.00  (CI: 0.95–1.05) | 0.47  (CI:0.44–0.51) | 0.35  (CI: 0.33–0.36) | 0.94  (CI: 0.89–0.97) |
| Ⅰ. Plus UAV data  (10%) | 0.46  (CI: 0.43–0.50) | 67.76  (CI: 64.91–  70.64) | 0.90  (CI: 0.92–1.00) | 0.47  (CI: 0.42–0.53) | 0.35  (CI: 0.33–0.37) | 0.94  (CI: 0.88–1.00) |
| Ⅱ. Plus UAV data  (30%) | 0.50  (CI: 0.45–0.53) | 63.96  (CI: 61.21–  67.48) | 0.90  (CI: 0.86–0.95) | 0.48  (CI: 0.41–0.55) | 0.34  (CI: 0.32–0.37) | 0.92  (CI: 0.86–0.98) |
| Ⅲ. Plus UAV data  (50%) | 0.52  (CI: 0.49–0.55) | 61.54  (CI: 58.96–  64.52) | 0.87  (CI: 0.83–0.91) | 0.49  (CI: 0.41–0.53) | 0.34  (CI: 0.32–0.36) | 0.90  (CI: 0.85–0.97) |
| Ⅳ. Plus UAV data  (70%) | 0.53  (CI: 0.51–0.55) | 60.18  (CI: 58.10–  62.14) | 0.85  (CI: 0.82–0.88) | 0.49  (CI: 0.45–0.55) | 0.33  (CI: 0.31–0.34) | 0.88  (CI: 0.83–0.91) |
| Ⅴ. Plus UAV data  (90%) | 0.53  (CI: 0.51–0.55) | 59.44  (CI: 57.21–  61.21) | 0.84  (CI: 0.81–0.86) | 0.49  (CI: 0.45–0.53) | 0.33  (CI: 0.31–0.34) | 0.87  (CI: 0.84–0.92) |

**Figure S3.4**

We compared six models with different numbers of training data inside the target forest management unit (FMU) to examine the impact of the number of Unmanned Aerial Vehicle (UAV)-based information on the accuracy of predicting Above-Ground Carbon (AGC) and Functional Group (FG) ratio. Materials and methods are described in Figure S3.3. *R*², RMSE and RRMSE are shown in graphs a and b for AGC, respectively, and in graphs c and d for FG ratio, respectively. First model was constructed using only tree inventory data outside the FMUs. The second to sixth models include 10%, 30%, 50%, 70%, and 90% of UAV-based information inside the FMUs, along with 90% of the data from outside the FMUs for training.

For both AGC and FG ratio, the prediction accuracy improved with increasing the number of UAV-based information. However, models with 70% and 90% of UAV-based information showed almost similar prediction accuracy. The accuracy to predict AGC with 90% (n = 300) of UAV-based information (*R*^2^ = 0.53) was comparable to the accuracy with approximately 70 points of tree inventory data within the FMUs (*R*^2^ = 0.54; Figure S3.2). On the other hand, the accuracy to predict FG ratio with 90% (n= 300) of UAV-based information (*R*^2^ = 0.49) was comparable to the accuracy with approximately 10 points of tree inventory data within the FMUs (*R*^2^ = 0.49; Figure S3.2).

**Supplementary 4: Overview of study plots and Forest Management Units (FMUs)**

The tree inventory plots used for Step 2 analyses in this study were established between 2013 and 2020 ([11, 12, 13, 14, 15]; Imai, unpublished data; Takeshige and Aoyagi, unpublished data; Sabah Forestry Department, unpublished data). Additional plots were established in 2023 and 2024 (Takeshige and Aoyagi, unpublished data). In total, 59 plots were used for Step 1–2 analyses. All plots were located in lowland or lower montane (<1200 m a.s.l.) mixed dipterocarp forest, the most common forest formation in Borneo. Plots were set up at least 100 m apart from the nearest plot.

These plots were distributed across four Forest Management Units (FMUs): Deramakot (5°14′–28′ N, 117°20′–38′ E; 551 km²), Tangkulap (5°18′–31′ N, 117°11′–22′ E; 276 km²), Segaliud Lokan (5°20′–27′ N, 117°23′–39′ E; 576 km²), and Ulu Segama–Malua (4°57′–50′ N, 117°51′–1′ E; 2,058 km²). Above‑ground carbon (AGC) information for these FMUs is presented in Table S4.1, and their logging history and current forest management practices are summarized in Table S4.2.

Table S4.1

Mean and standard deviation of above‑ground carbon (AGC; Mg ha⁻¹) reported for four Forest Management Units (FMUs) in Sabah, Malaysia, based on 2016 data [16].

| Region | Reported mean and standard deviation of AGC (Mg ha⁻¹) in 2016 [16]. |
| --- | --- |
| Deramakot FMU | 134.77±60.22 |
| Tangukulap FMU | 97.2±32.51 |
| Segaliud Lokan FMU | 67.98±28.29 |
| Ulu Segama-Malua FMU | 106.25±54.02 |

Table S4.2

Summary of historical logging activities and current forest management practices across four Forest Management Units (FMUs) in Sabah, Malaysia. Information includes past logging intensity, prohibition periods, and the adoption of Reduced Impact Logging (RIL) or conservation measures.

| Logging history | Current forest management |
| --- | --- |
| Deramakot FMU [17, 18] | |
| - Conventional high-intensity logging (1955-1989)  - Logging prohibited post-1989 | -RIL practices started in 1995 by Sabah Forestry Department  - 3rd forest management plan (2015-2024) |
| Tangkulap FMU [19] | |
| - Conventional logging until 2002  - Logging prohibited post-2002 | - Logging banned for forest regeneration by Sabah Forestry Department after 2002 |
| Segaliud Lokan FMU [20] | |
| - Conventional logging from 1995 to 1998 by private logging company | - RIL practices introduced after 1998 by private logging company |
| Ulu Segama-Malua FMU [21] | |
| - Large-scale, intensive logging (40 years, until mid-1990s)  - Re-logging began in 1999 | - Logging halted in 2007  - Protection agreement signed (Sabah Forestry Department, Yayasan Sabah, WWF-Malaysia)  - Current restoration and conservation efforts |

**Supplementary 5: Calculation methods of Above-Ground Carbon (AGC)**

First, we calculated Above-Ground Biomass (AGB) using the allometric equation in [22] (Equation S5.1, S5.2).

**Table S5**

Allometric equations used to estimate above‑ground biomass (AGB) and environmental stress index (E).

| No. | Index | Equation |
| --- | --- | --- |
| Equation S5.1 | AGB | $\exp\left[ -1.803-0.976E+0.976In\left( \rho\right)+2.673In\left( \mathrm{DBH} \right)-0.0299\left[ \mathrm{In}\left( \mathrm{DBH} \right) \right]^{2} \right]$ |
| Equation S5.2 | *E* | $\left( 0.178\times TS-0.938\times CWD-6.61\times PS \right)\times{10}^{-3}$ |

*E* represents a measure of environmental stress, *ρ* is species-specific wood density (g/cm³), DBH is the diameter at breast height (cm), *TS* is temperature seasonality and *CWD* is climate water deficit. We used the BIOMASS package to calculate *E* at the center of each plot [23]. Species-specific wood densities were sourced from various references (refer to [24] for detail). Above-ground biomass of each plot (Mg ha^–1^) was calculated by summing all measured trees within the plot and dividing it with plot area. AGC was calculated by multiplying AGB by 0.5. When species-level wood density data was unavailable, genus-level averages were used, and in cases lacking genus-level data, an overall average (0.58) was applied.

**Supplementary 6: Unmanned Aerial Vehicle (UAV) flight information and methods of image processing**

The UAV conducted automated flights based on a flight program created by UgCS 4.0.187 (SPH Engineering, Latvia, https://www.sphengineering.com/) and operated by UgCS for DJI (SPH Engineering, Latvia, https://www.sphengineering.com/). The forward and side overlap ratio were set 90% and 80%, respectively. Using the terrain follow function, we fixed the flight height at 100 m from the ground elevation provided by the digital elevation model of Shuttle Radar Topography Mission (SRTM). The captured images were processed using SfM (Structure for Motion) software Metashape Proffesional 1.7.2 (Agisoft, Russia, https://www.agisoft.com/), and generated orthomosaic photo, Digital Surface Model (DSM), and Digital Terrain Model (DTM). The DTM was created based on the ground points extracted by “Classify Ground Points” function. The Canopy Height Model (CHM) was derived by subtracting the DTM from the DSM, processed in R 4.1.1 [25]. The resolutions of the orthomosaic photo and CHM were approximately 2 cm and 5 cm, respectively.

**Supplementary 7: The acquired data and how they were used in this study**

**Table S7**

Utilization of data acquired across diverse regions and time periods in Step 1, together with the actual number of tree inventory records used for model development in Steps 1–2. The study sites in Deramakot and Tangkulap were completely distinct from one another in June and October 2023, as well as in September 2024.

| Data | Month-Year | Region | Step 1-1 (Model development) | Step 1-1 (Model application) | Step 1-2 (Model development) | Step 1-2 (Model application) |
| --- | --- | --- | --- | --- | --- | --- |
| Tree inventory data | Jun-23 | Deramakot & Tanngkulap |  |  | 〇  N = 16 |  |
|  | Jun-24 | Segaliud Lokan |  |  | 〇  N = 21 |  |
|  | Aug-24 | Ulu Segama-Malua |  |  | 〇  N = 19 |  |
| UAV-RGB images | Jun-23 | Deramakot & Tanngkulap | 〇 | 〇 | 〇 |  |
|  | Oct-23 | Deramakot & Tanngkulap | 〇 |  |  |  |
|  | Sep-24 | Deramakot & Tanngkulap | 〇 | 〇 |  | 〇 |
|  | Jun-24 | Segaliud Lokan |  | 〇 | 〇 | 〇 |
|  | Aug-24 | Ulu Segama-Malua |  | 〇 | 〇 | 〇 |
| Crown information | Jun-23 | Deramakot & Tanngkulap | 〇 |  |  |  |
|  | Oct-23 | Deramakot & Tanngkulap | 〇 |  |  |  |
|  | Sep-24 | Deramakot & Tanngkulap | 〇 |  |  |  |

**Supplementary 8: Dipterocarp crown delineation and training data preparation from Unmanned Aerial Vehicle (UAV)-RGB imagery**

First, candidates for dipterocarp trees were selected through visual inspection of the UAV-RGB imagery. We then confirm the species and delineate crown boundaries in the field (Figure S8). The species identification was conducted by direct observation of leaves, trunks, and fallen leaves, with verification by experts. We used Global Positioning System (GPS)-enabled iPads (Apple Inc, USA) and Adobe Photoshop software (Adobe Systems Incorporated, USA, https://www.adobe.com/) to delineate tree crowns.


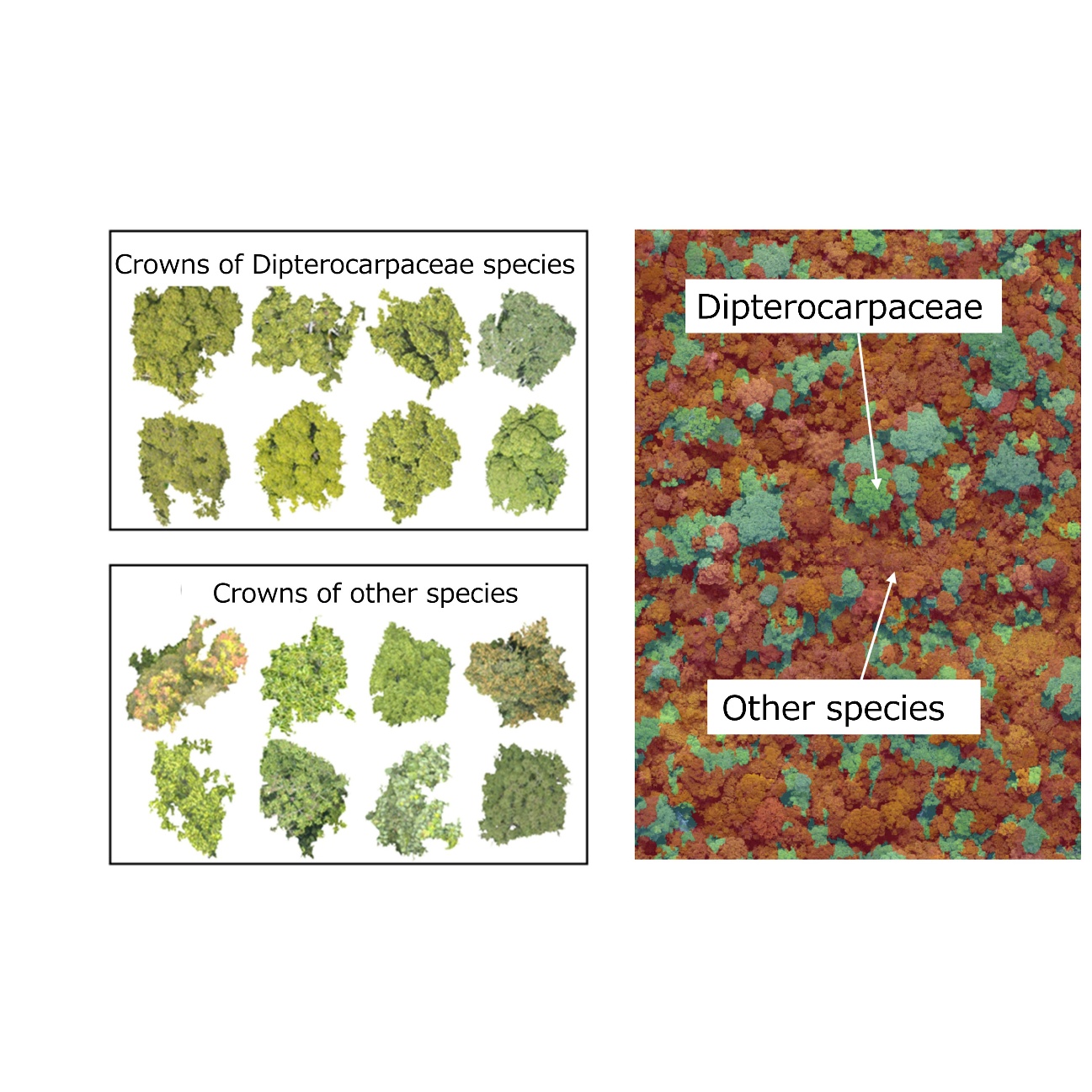


Figure S8

Examples of dipterocarp crown images (training data, left figure) in Unmanned Aerial Vehicle (UAV)-RGB imageries and the prediction of the deep learning model for a segmented canopy image (right figure). A single tree canopy was separated into multiple polygons (over-segmentation). Non-colored canopy and red-colored canopy in the right figure indicate dipterocarp canopy and other species’ canopy respectively.

**Supplementary 9: Unmanned Aerial Vehicle (UAV)-based canopy segmentation and training dataset preparation**

The tree canopy images and species name information in Photoshop were converted into vector data of tree crowns with class labels using QGIS 3.18 (QGIS Development Team, USA, https://qgis.org/). We then performed crown segmentation of UAV-RGB and Canopy Height Model (CHM) images using DF Scanner Pro 1.2.0 (DeepForest Technologies, Japan, https://deepforest-tech.co.jp/). Tropical tree species exhibit high diversity, with most trees being broadleaf species, which makes it challenging to segment individual trees accurately [7]. In some cases, a single tree canopy was segmented into multiple polygons. However, since the objective was to calculate dipterocarp crown area ratio within the plot, over-segmentation, where a single tree is represented by multiple polygons, was not an issue. The non-individual-based polygon data were overlaid with the species name vector data. If more than 70% of the area of a segmented canopy polygon overlapped with the species vector data, the corresponding species name (i.e., “Dipterocarpaceae” or “Others”) was assigned to the polygon. The training dataset included 1,948 samples of Dipterocarpaceae and 1,623 samples of other species, while the test dataset contained 85 samples of Dipterocarpaceae and 89 samples of other species.

Supplementary 10: The overall workflow of this study


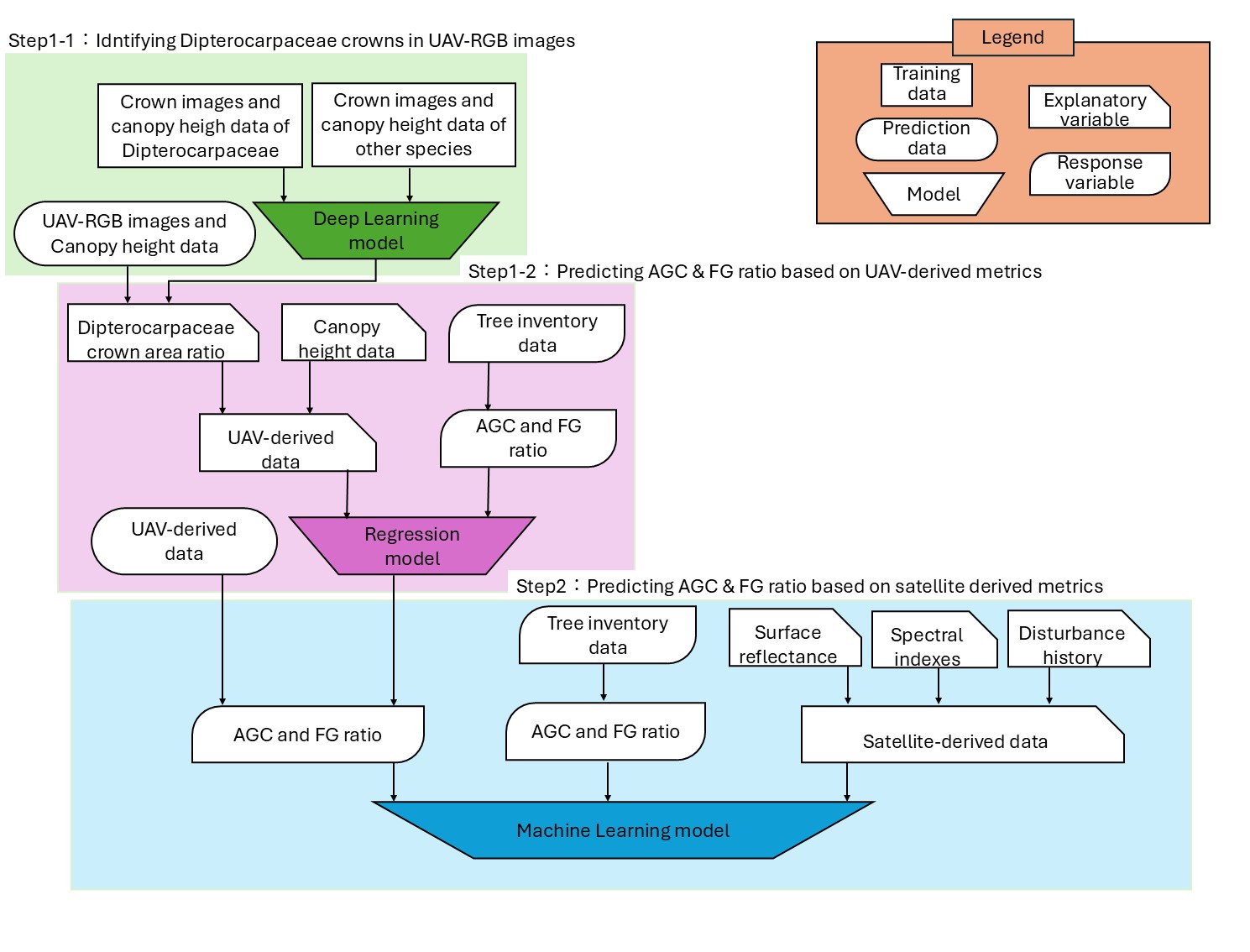


Figure S10

This study consists of three steps: 1-1) development of a deep learning model to detect Dipterocarpceae canopies, 2) development of regression models to predict Above-Ground Carbon (AGC) and Functional Group (FG) ratio using Unmanned Aerial Vehicle (UAV)-RGB image information and 3) development of a machine learning models to predict AGC and FG ratio using satellite image information.

**Supplementary 11: Calculation methods of variables related to canopy structure, gap, species composition**

First, we calculated variables related to canopy height, including the mean, median, standard deviation (SD), maximum of the Canopy Height Model (CHM), and the Vertical Distribution Ratio (VDR; [26]) within each plot (20-m radius). The VDR was calculated using Equation S11.1.

Second, we defined a gap as an area where the CHM was below 5 m and exceeded 1 m², and calculated the number of gaps, mean ratio of gap area to plot area, median ratio of gap area to plot area, SD ratio of gap area to plot area and maximum ratio of gap area to plot area. We also calculated Gap Shape Complexity Index (GSCI) using the equation S11.2 [27] for each plot and obtained mean of GSCI, median of GSCI, SD of GSCI and maximum of GSCI.

Finally, variables related to canopy class are the area ratio of dipterocarp canopy and that of other species’ canopy to the plot area.

**Table S11**

Structural indices derived from canopy height model (CHM) and crown geometry.

| No. | Index | Equation |
| --- | --- | --- |
| Equation S11.1 | VDR | $\left( \mathrm{CHM}_{\max} - \mathrm{CHM}_{\mathrm{median}} \right)／\mathrm{CHM}_{\max}$ |
| Equation S11.2 | GSCI | $Perimeter/\sqrt{4 \pi*Area}$ |

**Supplementary 12: Random plots distribution across Unmanned Aerial Vehicle (UAV)-RGB images**

**
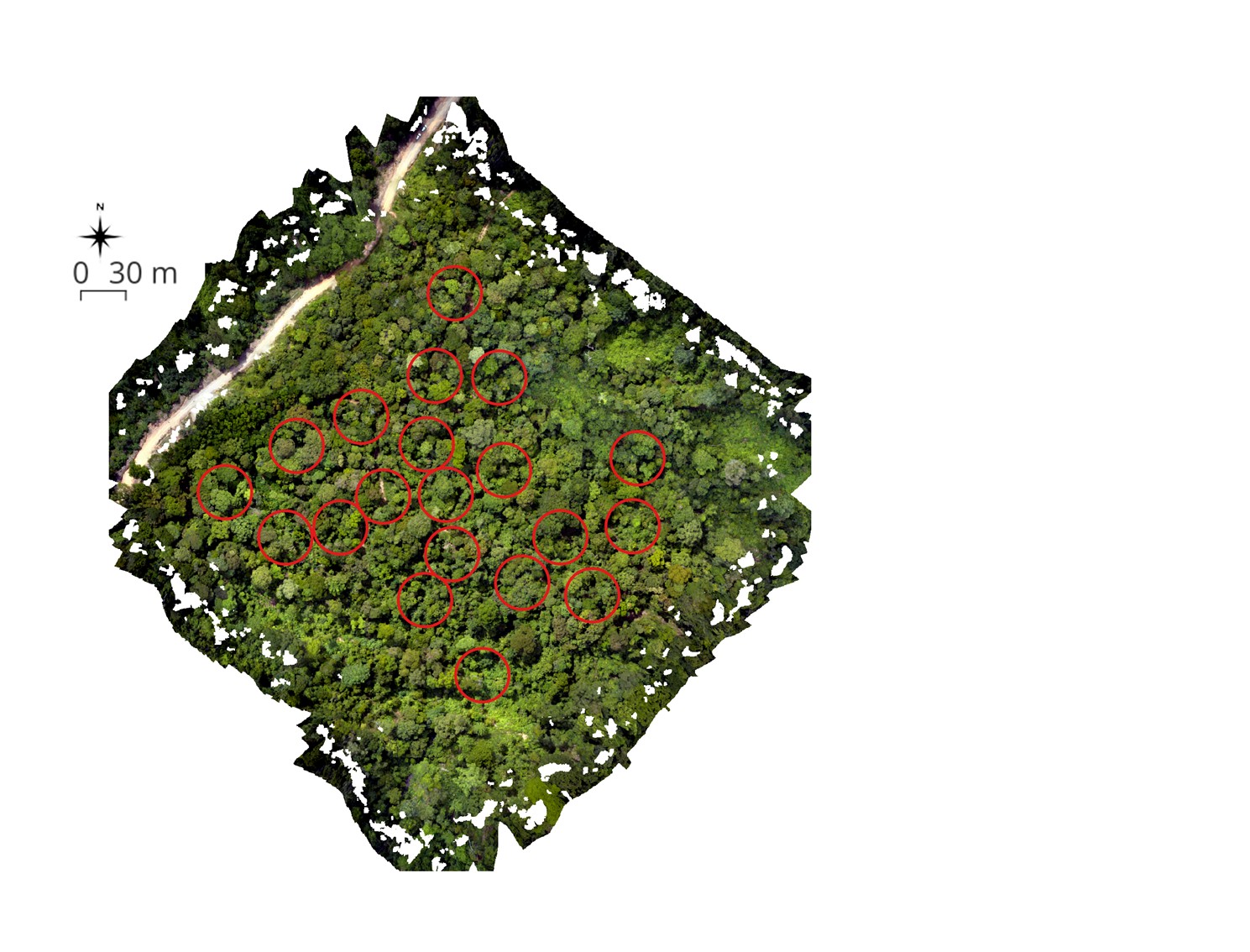
**

**Figure S12**

Twenty buffers were randomly created within forested areas of each of the forty‑eight UAV‑RGB images across four Forest Management Units (FMUs). Models estimating carbon stock and biodiversity index based on UAV‑derived metrics were applied to each buffer.

**Supplementary 13: Preprocessing of satellite images using Google Earth Engine (GEE)**

Preprocessing of satellite images was conducted using Google Earth Engine (GEE). We obtained satellite imagery corresponding to the year and location of each plot. For plots established between 2013 and 2020, we used Landsat 8 surface reflectance data (LANDSAT/LC08/C01/T1_SR). Composite images were generated by combining data from the target year, the preceding year, and the following year, with the median value representing each year. For plots established in 2024, we used both Landsat 8 and Landsat 9 imagery (LANDSAT/LC08/C02/T1_L2 and LANDSAT/LC09/C02/T1_L2). Topographic correction was performed using the SCS+C method [28]. Cloud removal was based on the quality assessment band, and images with cloud-to-land ratios greater than 80% or less than 10% were excluded. All imagery was projected to the coordinate reference system (CRS) EPSG:32650 — WGS 84 / UTM zone 50N.

**Supplementary 14: Equations for Selected Landsat Spectral Indices**

We calculated NDVI (Equation S14.1; [29]) using Near Infrared (NIR) and Red bands, Normalized Difference Water Index (NDWI; Equation S14.2; [30]) using Shortwave Infrared 1 (SWIR1) and NIR bands, Normalized Difference Soil Index (NDSI; Equation S14.3; [31]) using Green and SWIR1 bands, Enhanced Vegetation Index (EVI; Equation S14.4; [32]) using NIR, Red, and Blue bands, and Normalized Burn Ratio (NBR; Equation S14.5; [33]) using NIR and SWIR bands from Landsat imagery.

**Table S14**

Spectral indices and their corresponding equations used in this study.

| No. | Index | Equation |
| --- | --- | --- |
| Equation S14.1 | NDVI | $(NIR-Red)/(NIR+Red$) |
| Equation S14.2 | NDWI | $(SWIR1-NIR)/(SWIR1+NIR)$ |
| Equation S14.3 | NDSI | $(Green-SWIR1)/(Green+SWIR1)$ |
| Equation S14.4 | EVI | $(2.5\times\left( NIR-Red \right))/(NIR-6\times Red-7.5\times Blue+1)$ |
| Equation S14.5 | NBR | $(NIR-SWIR)/(NIR+SWIR)$ |

**Supplementary 15: Disturbance detection and Normalized Burn Ration (NBR)-based indices calculation using LandTrendr**

Abrupt changes in NBR exceeding specific thresholds were identified as disturbances in LandTrendr [34]. The threshold criteria included a magnitude of change greater than 30 inside four years, a pre-disturbance NBR value of ≥300, and a patch size of ≥5 pixels. Subsequently, the following indices were calculated for pixels inside 20-m radius plots. The mean values of 15 randomly sampled points inside each plot were used as representative values. If no disturbance was observed after 1980, yod was set to 1980, while the other variables were set as zero.

**Supplementary 16: Adjustment of random forest predictions**

To mitigate biases inherent in the random forest algorithm, the final Above-Ground Carbon (AGC) and Functional Group (FG) ratio predictions were adjusted during cross-validation. This adjustment involved multiplying the predicted AGC and FG ratio by the slope derived from the Type Ⅱ　regression between the predicted and observed values inside each cross-validation fold and adding the intercept of the regression equation. We used Type Ⅱ regression because unlike Type Ⅰ regression,　it does not reduce the variation in predictions.

**Supplementary 17: Cross validation procedures for the satellite analyses**

**
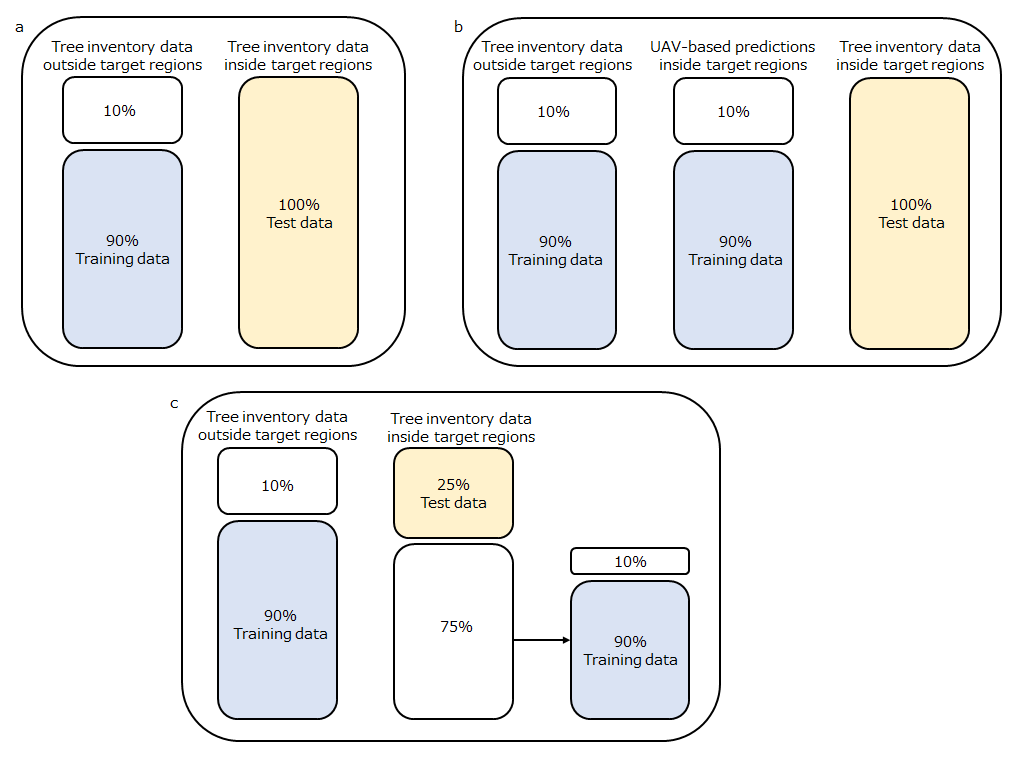
**

Figure S17

Information of cross validation of the models to predict Above-Ground Carbon (AGC) and Functional Group (FG) ratios in four Forest Management Units (FMUs). In Model 1 (a), 90% of the tree inventory data from outside the target regions was randomly selected for training, and all tree inventory data inside the target regions was used for test. In Model 2 (b), 90% of the training data came from tree inventory data outside the target regions, along with Unmanned Aerial Vehicle (UAV)-based predictions from inside the target regions, and all tree inventory data inside the target regions was used for test. In each iteration, we established regression models to predict AGC and FG ratio based on UAV RGB data using subsets (25%) of all plot data and removed the plots used to established the models from test data so that no data were used simultaneously for both training the regression model in Step 1-2 and testing. In Model 3 (c), 25% of the tree inventory data from each target region was set aside as test data. For training, 90% of the remaining 75% of the tree inventory data from inside the target regions (i.e., 67.5% of the total data from inside the target regions), along with 90% of the tree inventory data randomly selected from outside the target regions, was used. This process was repeated four times to ensure that all data from the target regions was used as test data once. *R*², Root Mean Square Error (RMSE) and Relative RMSE (RRMSE) were calculated based on predicted and observed values, with the entire process repeated 100 times to compute the mean and 95% Confidence Intervals (CIs) for each model. In the figure, blue and orange represent the training and test data, respectively.

**Supplementary References**

1. Paneque-Gálvez, J., McCall, M.K., Napoletano, B.M., Wich, S.A., & Koh, L.P. (2014.) Small drones for community-based forest monitoring: An assessment of their feasibility and potential in tropical areas. *Forests*, *5*(6), 1481-1507. <https://doi.org/10.3390/f5061481>
2. Yao, H., Qin, R., & Chen, X. (2019). Unmanned aerial vehicle for remote sensing applications—A review. *Remote sensing*, 11(12), 1443. <https://doi.org/10.3390/rs11121443>
3. Nex, F., & Remondino, F. (2014). UAV for 3D mapping applications: A review. *Applied geomatics*, *6*, 1-15. <https://doi.org/10.1007/s12518-013-0120-x>
4. Onishi, M., & Ise, T. (2021). Explainable identification and mapping of trees using UAV RGB image and deep learning. *Scientific reports*, *11*(1), 903. https://doi.org/10.1038/s41598-020-79653-9
5. Matese, A. et al. (2015). Intercomparison of UAV, aircraft and satellite remote sensing platforms for precision viticulture. *Remote Sensing*, *7*(3), 2971–2990. https://doi.org/10.3390/rs70302971
6. Li, Z. et al. (2019). Remote estimation of mangrove aboveground carbon stock at the species level using a low-cost unmanned aerial vehicle system. *Remote Sensing*, *11*(9), 1018. https://doi.org/10.3390/rs11091018
7. Zhang, J. et al. (2022). Drone-acquired data reveal the importance of forest canopy structure in predicting tree diversity. *Forest Ecology and Management*, *505*, 119945. https://doi.org/10.1016/j.foreco.2021.119945
8. Diez, Y. et al. (2021). Deep learning in forestry using uav-acquired rgb data: A practical review. *Remote Sensing*, *13*(14), *2837*. https://doi.org/10.3390/rs13142837
9. Adão, T. Et al. (2017). Hyperspectral Imaging: A Review on UAV-Based Sensors, Data Processing and Applications for Agriculture and Forestry. Remote Sensing, 9(11)*,* 1110. <https://doi.org/10.3390/rs9111110>
10. Tao, W., Chen, Y., Huang, L., Jing, K., & Cheng, Z. (2024). Stability Detection of Canopy RGB Images for Different Underlying Surfaces Based on SVM. *Atmosphere*, *15*(8), 943. https://doi.org/10.3390/atmos15080943
11. Fujiki, S., Aoyagi, R., Tanaka, A., Imai, N., Kusma, A., Kurniawan, Y., Lee, Y., Sugau, J., Pereira, J., Samejima, H., Kitayama, K. (2016). Large-Scale Mapping of Tree-Community Composition as a Surrogate of Forest Degradation in Bornean Tropical Rain Forests. *Land*, *5*(4), 45. https://doi.org/10.3390/land5040045
12. Kitayama, K. et al. (2018). Biodiversity observation for land and ecosystem health (BOLEH): A robust method to evaluate the management impacts on the bundle of carbon and biodiversity ecosystem services in tropical production forests. *Sustainability*, *10*(11), 4224. https://doi.org/10.3390/su10114224
13. Miyamoto, K., Aiba, S., Aoyagi, R., & Nilus, R. (2021). Effects of El Niño drought on tree mortality and growth across forest types at different elevations in Borneo. *Forest Ecology and Management*, *490*, 119096. <https://doi.org/10.1016/j.foreco.2021.119096>
14. Yano, S. et al. (2021). Effects of logging on landscape-level tree diversity across an elevational gradient in Bornean tropical forests. *Global Ecology and Conservation*, *29*, e01739. <https://doi.org/10.1016/j.gecco.2021.e01739>
15. Takeshige, R. et al. (2023). Influences of fern and vine coverage on the above-ground biomass recovery in a Bornean logged-over degraded secondary forest.  *Journal of Forest Research*, *28*(4), 260-270. https://doi.org/10.1080/13416979.2023.2187682
16. Asner, G. P. et al. (2018). Mapped aboveground carbon stocks to advance forest conservation and recovery in Malaysian Borneo. *Biological Conservation*, *217*, 289–310. <https://doi.org/10.1016/j.biocon.2017.10.020>
17. Lagan, P., Mannan, S., & Matsubayashi, H. (2007). *Sustainable use of tropical forests by reduced-impact logging in Deramakot Forest Reserve, Sabah, Malaysia*. Springer, Tokyo, Japan, 414-421.
18. Sabah Forestry Department, (2014). 3rd Forest Management Plan. <https://deramakot.sabah.gov.my/images/pdf/pub/3rd_FMP_Deramakot.pdf>. Accessed 28 March 2025.
19. Ong, R.C., Langner, A., Imai, N., & Kitayama, K. (2013).  Management history of the study sites: The Deramakot and Tangkulap forest reserves. In *Co-Benefits of Sustainable Forestry: Ecological Studies of a Certified Bornean Rain Forest*. Kitayama, K., Ed. Springer, Tokyo, Japan, 1–22.
20. SIRIM QAS International Sdn. Bhd. (2018). PUBLIC SUMMARY SURVEILLANCE 2 AUDIT (3 RD CYCLE) ON SEGALIUD LOKAN FOREST RESERVE MANAGEMENT UNIT FOR FOREST MANAGEMENT CERTIFICATION. <https://www.sirim-qas.com.my/wp-content/uploads/2020/09/Public-Summary-FMC-of-Segaliud-Lokan-Forest-Management-Unit-2nd-Surveillance-Audit-3rd-Cycle_v1.0.pdf>. Accessed 28 March 2025.
21. Reynolds, G., Payne, J., Sinun, W., Mosigil, G., & Walsh, R.P.D. (2011). Changes in forest land use and management in Sabah, Malaysian Borneo, 1990-2010, with a focus on the Danum Valley region. *Philosophical Transactions of the Royal Society B: Biological Sciences*, *366*(1582), 3168-3176. https://doi.org/10.1098/rstb.2011.0154
22. Chave, J. et al. (2014). Improved allometric models to estimate the aboveground biomass of tropical trees. *Global Change biology*, *20*(10), 3177-3190. https://doi.org/10.1111/gcb.12629
23. Réjou‐Méchain, M., Tanguy, A., Piponiot, C., & Chave, J., Hérault, B. (2017). BIOMASS : an R package for estimating above-ground biomass and its uncertainty in tropical forests.forests. *Methods in Ecology and Evolution*, *8*(9), 1163-1167. https://doi.org/10.1111/2041-210X.12753
24. Imai, N. et al. (2014). Tree community composition as an indicator in biodiversity monitoring of REDD+. Forest Ecology and Management, *313*, 169–179. https://doi.org/10.1016/j.foreco.2013.10.041
25. R-Core-Team. (2021). R: A Language and Environment for Statistical Computing. R Foundation for Statistical Computing, Vienna. https://www.R-project.org
26. Goetz, S., Steinberg, D., Dubayah, R., & Blair, B. (2007). Laser remote sensing of canopy habitat heterogeneity as a predictor of bird species richness in an eastern temperate forest, USA. *Remote Sensing of Environment*, *108*(3), 254-263. https://doi.org/10.1016/j.rse.2006.11.016
27. Patton, D.R. (1975). A diversity index for quantifying habitat “edge”. *Wildlife Society Bulletin (1973-2006)*, *3*(4), 171-173.
28. Soenen, S.A., Peddle, D.R., & Coburn, C.A. (2005). SCS+C: A modified sun-canopy-sensor topographic correction in forested terrain. *IEEE Transactions on geoscience and remote sensing*, *43*(9), 2148-2159. https://doi.org/10.1109/TGRS.2005.852480
29. Rouse, J.W., Haas, R.H., Schell, J.A., Deering, D.W., & Harlan, J.C. (1974). *Monitoring the vernal advancement and retrogradation (green wave effect) of natural vegetation* (No. E75-10354).
30. Gao, B. C. (1996). NDWI—A Normalized Difference Water Index for Remote Sensing of Vegetation Liquid Water From Space. *Remote Sensing of Environment*, *58*(3), 257-266. <https://doi.org/10.1016/S0034-4257(96)00067-3>
31. Takeuchi, W., & Yasuoka, Y. (2005). Development of normalized vegetation, soil and water indices derived from satellite remote sensing data. *Journal of the Japan society of photogrammetry and remote sensing*, *43*(6), 7-19. <https://doi.org/10.4287/jsprs.43.6_7>
32. Huete, A. et al. (2002). Overview of the radiometric and biophysical performance of the MODIS vegetation indices. *Remote sensing of environment*, *83*(1-2), 195-213. <https://doi.org/10.1016/S0034-4257(02)00096-2>
33. Miller, J.D., & Thode, A.E. (2007). Quantifying burn severity in a heterogeneous landscape with a relative version of the delta Normalized Burn Ratio (dNBR). *Remote Sensing of Environment,* *109*(1), 66–80. https://doi.org/10.1016/j.rse.2006.12.006
34. Gorelick, N. et al. (2017). Google Earth Engine: Planetary-scale geospatial analysis for everyone. *Remote Sensing of Environment*, 202, 18–27. https://doi.org/10.1016/j.rse.2017.06.031
